# A Forward-Design Approach to Increase the Production of Poly-3-Hydroxybutyrate in Genetically Engineered *Escherichia coli*

**DOI:** 10.1371/journal.pone.0117202

**Published:** 2015-02-20

**Authors:** Richard Kelwick, Margarita Kopniczky, Iain Bower, Wenqiang Chi, Matthew Ho Wai Chin, Sisi Fan, Jemma Pilcher, James Strutt, Alexander J. Webb, Kirsten Jensen, Guy-Bart Stan, Richard Kitney, Paul Freemont

**Affiliations:** 1 Centre for Synthetic Biology and Innovation, South Kensington Campus, London, United Kingdom; 2 Department of Medicine, South Kensington Campus, London, United Kingdom; 3 Department of Life Sciences, South Kensington Campus, London, United Kingdom; 4 Department of Bioengineering, Imperial College London, South Kensington Campus, London, United Kingdom; Tsinghua University, CHINA

## Abstract

Biopolymers, such as poly-3-hydroxybutyrate (P(3HB)) are produced as a carbon store in an array of organisms and exhibit characteristics which are similar to oil-derived plastics, yet have the added advantages of biodegradability and biocompatibility. Despite these advantages, P(3HB) production is currently more expensive than the production of oil-derived plastics, and therefore, more efficient P(3HB) production processes would be desirable. In this study, we describe the model-guided design and experimental validation of several engineered P(3HB) producing operons. In particular, we describe the characterization of a hybrid *phaCAB* operon that consists of a dual promoter (native and J23104) and RBS (native and B0034) design. P(3HB) production at 24 h was around six-fold higher in hybrid *phaCAB* engineered *Escherichia coli* in comparison to *E. coli* engineered with the native *phaCAB* operon from *Ralstonia eutropha* H16. Additionally, we describe the utilization of non-recyclable waste as a low-cost carbon source for the production of P(3HB).

## Introduction

Conventional oil-derived polyolefin plastics exhibit useful characteristics that have extensive commercial applications. However, the accumulation of plastics in the environment and the non-renewable source of polyolefin plastics have stimulated interest in sustainable sources of plastic production. Biopolymers, such as poly-3-hydroxybutyrate (P(3HB)), are produced as a carbon store in an array of organisms and exhibit characteristics which are similar to oil-derived plastics [1,2]. Furthermore, P(3HB) has the added advantages of biodegradability, biocompatibility and, based upon several life cycle analyses, P(3HB) production is more environmentally sustainable than polyolefin plastic production [1,3,4]. Despite these advantages, P(3HB) production is currently more expensive than the production of oil-derived plastics, and therefore, more efficient P(3HB) production processes would be desirable [5].

Genetic engineering approaches—in which the P(3HB)-producing operon, *phaCAB*, is cloned into *Escherichia coli*—have pioneered the industrial production of P(3HB) [6]. More recently, synthetic biology approaches involving the rational engineering of the *phaCAB* operon [7], and metabolic engineering strategies [8] have continued to increase the efficiency of P(3HB) production. In this paper we report on the forward-design and experimental validation of a rationally engineered *phaCAB* operon with a hybrid promoter design. Additionally, we describe the utilization of non-recyclable waste as a low-cost carbon source for the production of P(3HB).

## Results and Discussion

**Fig 1:**
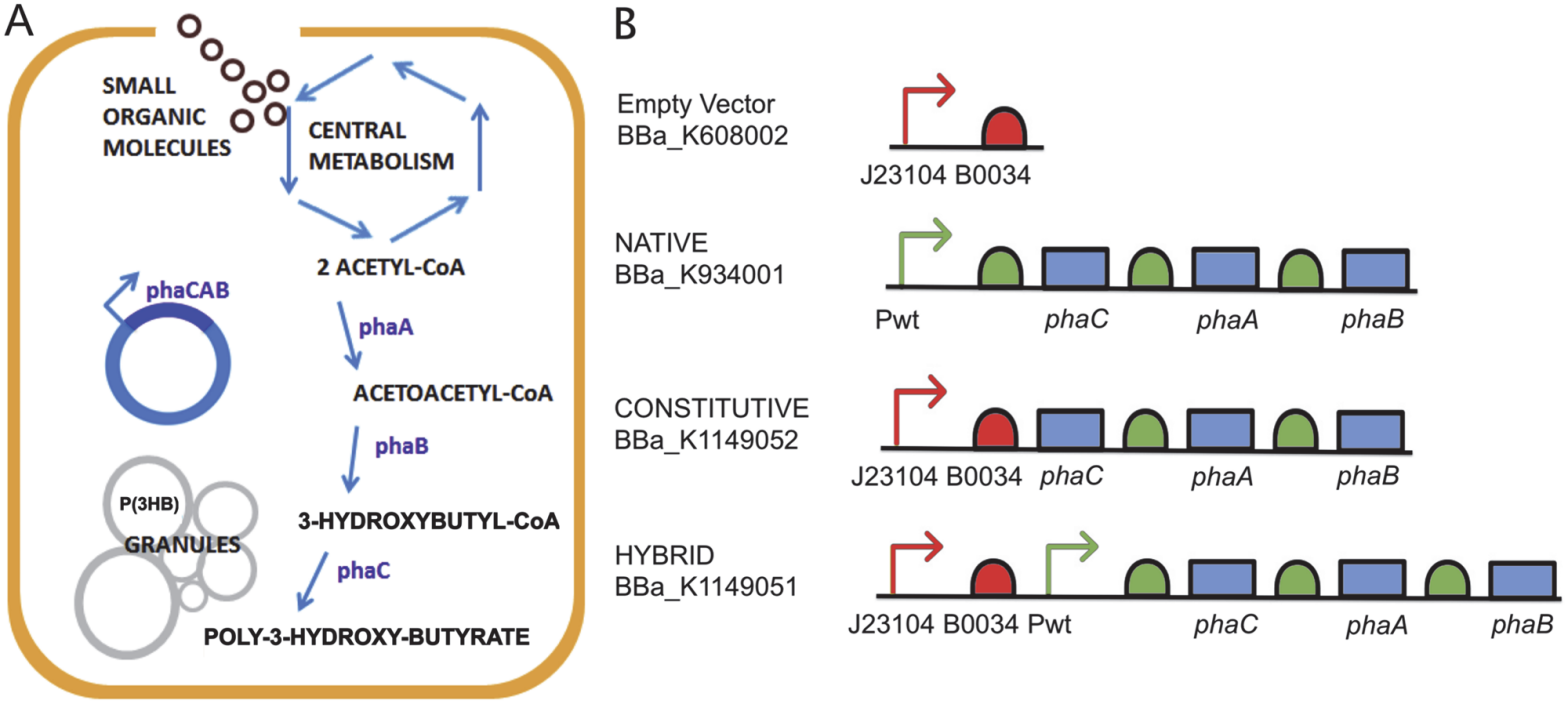
*phaCAB* pathway and constructs. (**A**) Schematic of poly-3-hydroxybutyrate (P(3HB)) production via the *phaCAB* operon pathway. (**B**) The constructs used in this study. Abbreviations: Pwt (wildtype promoter; green arrow), J23104 (Anderson constitutive promoter, BBa_J23104; red arrow), B0034 (ribosomal binding site, BBa_B0034; red half-circle), *phaC* (PHA synthase), *phaA* (3-ketothiolase) and *phaB* (acetoacetyl-CoA reductase). Green half-circles denote native ribosomal binding sites. Construct symbols are based on the Synthetic Biology Open Language Visual (SBOLv) v1.0.0 guidelines [21].

The *phaCAB* operon from *Ralstonia eutropha* H16, is the most extensively studied P(3HB) synthesis operon [2,9]. It consists of three enzymes, which through a multi-stage enzymatic process generate P(3HB) inside the cell from the central metabolite acetyl-CoA [1]. The process is shown in Fig. 1A and is briefly summarised here. Firstly, PhaA (3-ketothiolase) combines two molecules of acetyl-CoA to form acetoacetyl-CoA. Next, PhaB (acetoacetyl-CoA reductase) reduces acetoacetyl-CoA to form (R)-3-hydroxybutyl-CoA, which is then polymerised by PhaC (PHA synthase) to form poly-3-hydroxybutyrate P(3HB). The *phaCAB* operon from *R*. *eutropha* H16 was originally cloned into *E*. *coli* in the late 1980s [2]. More recently the Tokyo Tech 2012 iGEM team (http://2012.igem.org/Team:Tokyo_Tech) cloned, characterised and standardized the native *phaCAB* operon (BBa_K934001) into a biobrick-compatible format for the synthetic biology community.

In order to increase P(3HB) production, this study used a forward-design approach that is based upon the engineering principles of synthetic biology [10]. Several *phaCAB* operons were rationally engineered (Fig. 1B). The constitutive *phaCAB* operon (BBa_K1149052) was designed such that the native promoter and RBS were replaced with the constitutive promoter J32104 from the Anderson collection [11] and the RBS B0034. The hybrid *phaCAB* operon design (BBa_K1149051) was constructed in parallel to the constitutive operon and was noted for its dual promoter and RBS combinations (Fig. 1B). Design considerations were based upon modeling simulations of the PhaCAB pathway in engineered *E*. *coli*. The model was comprised of the glycolysis pathway, the tricarboxylic acid (TCA) cycle and the *phaCAB* synthetic pathway (S1 Supporting Information; S2 Supporting Information). These pathways were coupled in order to reflect the metabolic flux [12] of several metabolites and species between them and to provide a qualitative indication of their influence on P(3HB) production.

**Fig 2:**
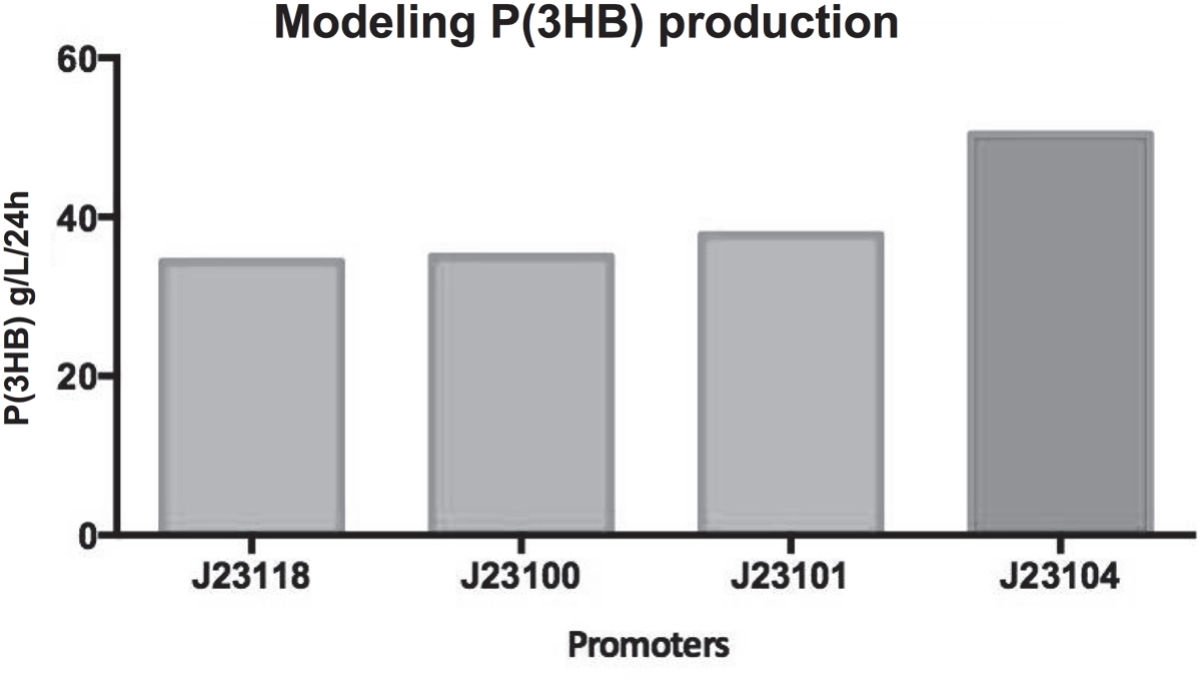
Simulated P(3HB) production in *phaCAB*-engineered *E*. *coli*. In order to simulate P(3HB) production in *phaCAB*-engineered *E*. *coli*, a P(3HB) synthesis model was constructed using the Simbiology toolbox of Matlab. Using this model the flux of several metabolites and species were simulated in order to identify aspects of the system that could be selectively tuned to increase the production of P(3HB). From these analyses, several novel *phaCAB* operons were designed. These data show the simulated P(3HB) production across several different *phaCAB* operon designs, where *phaCAB* expression is under the control of the indicated Anderson constitutive promoters.

A sensitivity analysis of species within the synthetic pathway revealed that increasing expression of *phaB* would be critical for directing the reaction flow towards the end product—an increase in P(3HB) production (S1 Supporting Information; S2 Supporting Information). In order to increase expression of *phaB*, further simulations predicted that of the several simulated *phaCAB* operon designs, an operon that incorporates the constitutive promoter, J23104, would result in the greatest increase in P(3HB) production (Fig. 2).

**Fig 3:**
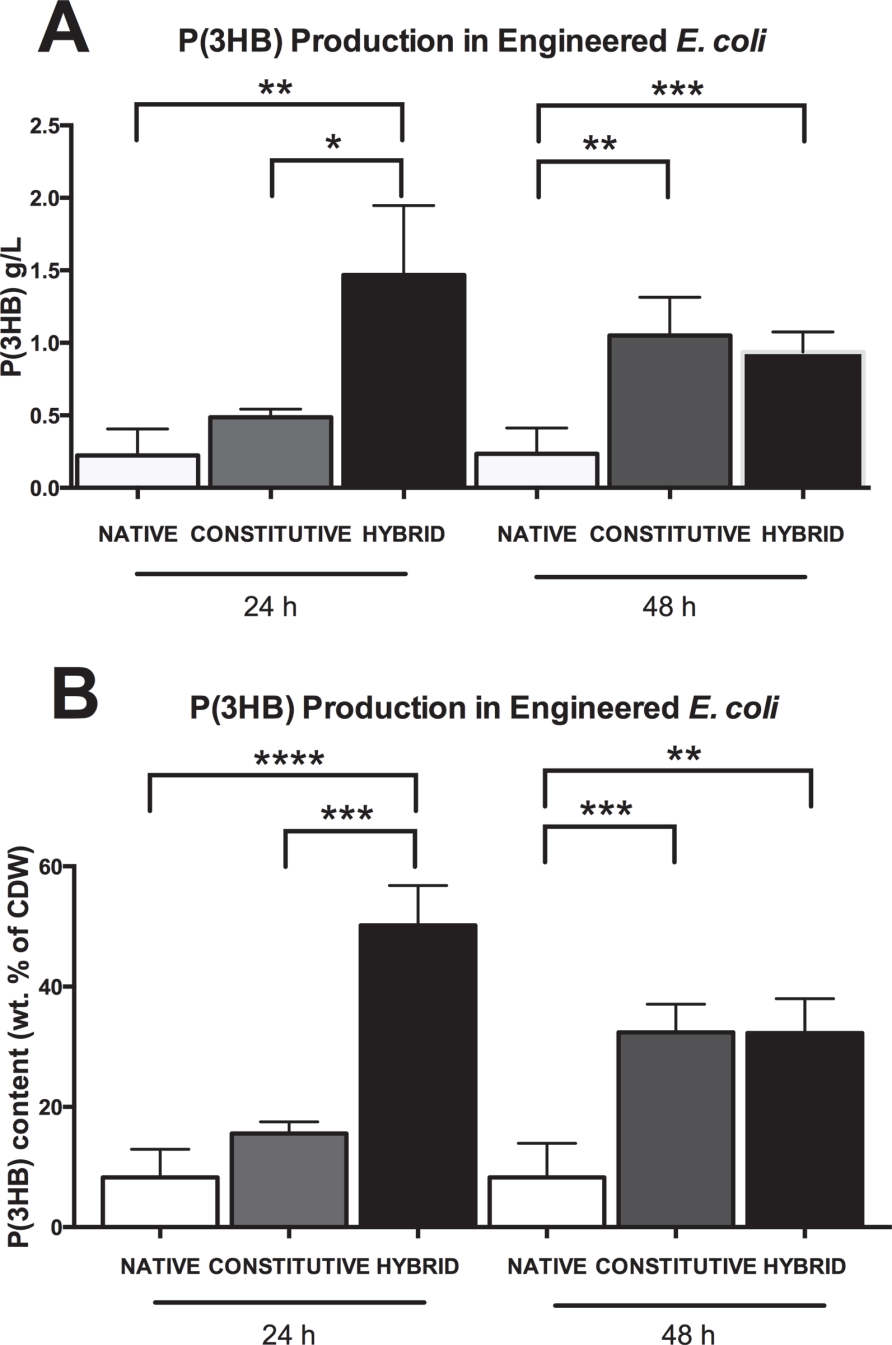
P(3HB) production in *phaCAB*-engineered *E*. *coli*. *E*. *coli* MG1655 transformed with empty vector, native, constitutive or hybrid *phaCAB* constructs were cultured in 1 liter LB media, supplemented with 3% glucose (w/v) for 24 hours or 48 hours. P(3HB) was purified from these cultures and measured as **(A)** P(3HB) production (g/L) and **(B)** P(3HB) content (weight [wt.] % of cell dry weight [CDW]). Data represent the mean +/- the standard deviation of three independent experiments. Student t-test, *P<0.05, **P <0.01, ***P <0.001 and ****P <0.0001.

To experimentally validate our forward-design approach, *E*. *coli* MG1655 carrying either the empty vector (BBa_K608002), native *phaCAB* (BBa_K934001), constitutive *phaCAB* (BBa_K1149052) or hybrid *phaCAB* (BBa_K1149051) operons were cultured with Lysogeny broth (LB) media, supplemented with 3% glucose (w/v) for either 24 or 48 hours. P(3HB) was purified from each of the engineered populations using sodium hypochlorite and were weighed for comparison (Fig. 3A). Average P(3HB) production at 24 hours was around two-fold higher in constitutive *phaCAB*-engineered *E*. *coli* (0.49 g/L S.D. ± 0.06) compared to native *phaCAB* (0.22 g/L S.D. ± 0.18), while average P(3HB) production was six-fold higher in hybrid *phaCAB*-engineered *E*. *coli* (1.47 g/L S.D. ± 0.48). At 48 hours, P(3HB) production was around three-fold higher in constitutive *phaCAB*-engineered *E*. *coli* (1.05 g/L S.D. ± 0.26) and hybrid *phaCAB*-engineered *E*. *coli* (0.94 g/L S.D. ± 0.14), compared to native *phaCAB* (0.30 g/L S.D. ± 0.14) (Fig. 3A). In hybrid *phaCAB*-engineered *E*. *coli* we observed a decrease in P(3HB) content, expressed as a percentage of the cell dry weight (CDW) from 50% at 24 hours to 32% at 48 hours (Fig. 3B). Similar observations have been previously reported, and is understood as a consequence of competition for cellular metabolites between cell growth and P(3HB) production [13–15]. The empty vector-engineered *E*. *coli* did not produce detectable levels of P(3HB).

Qualitative analysis of Nile Red stained *E*. *coli* also confirmed that the constitutive and hybrid *phaCAB* operon designs increased P(3HB) production (S3 Supporting Information). It is likely that the dual promoter and RBS design (Fig. 1B) of the hybrid system results in a higher level of mRNA transcript production and/or ribosome recruitment and thus an increase in the expression of the PhaCAB enzymes. qRT-PCR analysis, confirmed that both the hybrid and constitutive *phaCAB* operon designs result in higher levels of *phaCAB* transcripts than that of the native *phaCAB* operon (S6 Supporting Information).

Interestingly, as shown in Fig. 3A, P(3HB) production from the hybrid operon at 24 h was greater than the combined P(3HB) production of the native and constitutive operons. Together, these data suggest that the hybrid promoter performs a multiplicative, rather than an additive combination of the native and constitutive promoter designs. Unlocking the design rules of the hybrid promoter may have applicability that extends beyond the *phaCAB* operon. For instance, Li *et al*. 2012, suggest that engineered promoter clusters may be a useful metabolic engineering approach for increasing metabolite production. Additionally, model-guided optimisation of the PhaCAB pathway led to the generation of the constitutive operon design, which in combination with the hybrid promoter, represent an emerging family of rationally engineered P(3HB)-producing operons.

**Fig 4:**
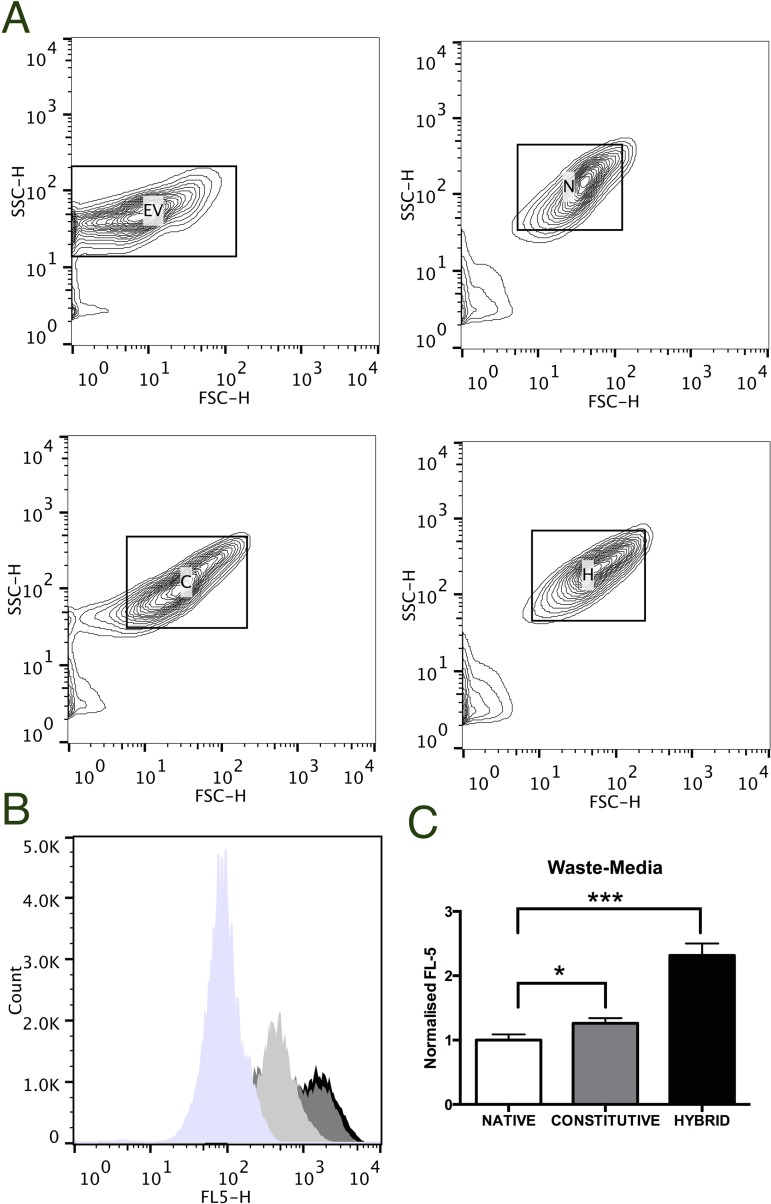
Flow cytometry analysis of P(3HB) production in *phaCAB*-engineered *E*. *coli* from waste-media cultures. *E*. *coli* MG1655 transformed with either empty vector [EV], native [N], constitutive [C] or hybrid [H] *phaCAB* constructs were cultured in 5 ml of waste-media for 36 h at 37°C. P(3HB) content was assessed via flow cytometry analysis of Nile Red staining. (**A**) Representative forward scatter (FSC) and side scatter (SSC) contour plots. (**B**) Representative histogram (FL-5). (**C**) Normalized fluorescence of Nile Red stained *phaCAB*-engineered *E*. *coli*, from three independent experiments. Error bars, +/- the standard deviation. Student t-test, *P<0.05 and ***P <0.001.

The broader project aim was to exploit non-recyclable waste as a low-cost carbon source that could be utilized by engineered *E*. *coli* tasked with the *de novo* synthesis of P(3HB). Industrial processing of non-recyclable mixed waste into solid and liquefied fractions of mixed cellulosic (~80%) and plastic (~20%) streams is currently possible [16]. However, these processes could be made more efficient through the use of natural and genetically engineered organisms that can both degrade and use these waste streams as a low-cost carbon source for the production of bioplastics. Beneficially, this approach may also result in the diversion of non-recyclable waste away from environmentally damaging activities such as landfill or incineration [3]. To this end, *phaCAB*-engineered *E*. *coli* were cultured in waste-media, which is analogous to industrially processed liquefied cellulosic waste; and from these cultures, P(3HB) was purified according to a modified sodium hypochlorite protocol, where waste-media cultures were filtered prior to centrifugation to remove residual waste material. Purified P(3HB) from each culture was depolymerized with recombinant phaZ1 into monomeric 3-hydroxybutyrate (3HB), which could then be detected as a yellow colour change when these samples were analysed with the β-Hydroxybutyrate colorimetric assay kit (S4 Supporting Information). PhaZ1-treated P(3HB) from *E*. *coli* carrying either the empty vector or native *phaCAB* operon resulted in a negligible color change, suggesting that little or no P(3HB) was produced in those cultures. Whilst, PhaZ1-treated P(3HB) from *E*. *coli* carrying either the constitutive *phaCAB* or hybrid *phaCAB* operon, depolymerized into 3HB, as indicated by a detectable, yellow color change. Additionally, flow cytometry analysis of Nile Red stained, [17,18] *phaCAB*-engineered *E*. *coli* from waste-media cultures, confirmed an increase in P(3HB) content (Fig. 4).

In conclusion, our study used a forward-design approach to rationally engineer novel *phaCAB* operon designs that were experimentally validated to increase the production of P(3HB) in genetically engineered *E*. *coli*. Additionally, we have described the utilization of non-recyclable waste as a low-cost carbon source for the production of P(3HB). With further development, these strategies, in combination with additional synthetic biology approaches [7,8], will further increase the efficiency and commercial viability of P(3HB) production.

## Materials and Methods

### Modeling

The P(3HB) synthesis model was constructed and simulated using the Simbiology toolbox of Matlab. The model is comprised of the glycolysis pathway, the tricarboxylic acid (TCA) cycle and the *phaCAB* synthetic pathway. Full details are provided as supplementary information (S1 Supporting Information).

### Construct assembly

Empty vector (BBa_K608002), and native *phaCAB* (BBa_K934001) constructs were sourced from the 2013 distribution of the iGEM Registry of Standard Biological Parts (partsregistry.org). The constitutive *phaCAB* operon (BBa_K1149052) was generated via PCR, with the native *phaCAB* operon (BBa_K934001) as the template. Primers Pha_Fw 5′-cgcttctagagatggctactgggaaaggagccg-3′ and BBa_G1005 5′-gtttcttcctgcagcggccgctactagta-3′, were utilised to generate a PCR product containing the *phaCAB* operon but excluding the native promoter and RBS. The PCR product was cloned into the destination vector (BBa_K608002) to generate the final constitutive *phaCAB* operon construct. The hybrid *phaCAB* (BBa_K1149051) construct was generated in parallel to the constitutive *phaCAB* operon. The entire native *phaCAB* operon including the native promoter and RBS was cloned into the destination vector (BBa_K608002) to generate the final hybrid *phaCAB* operon construct. All constructs were generated using the iGEM submission backbone, pSB1C3 and all restriction digests utilized the standard iGEM prefix and suffix restriction sites. Full sequences are provided as supplementary information (S5 Supporting Information).

### P(3HB) production

*E*. *coli* strain MG1655 carrying either empty vector, native *phaCAB*, constitutive *phaCAB* or hybrid *phaCAB* constructs were grown in LB media supplemented with 34 μg/mL Chloramphenicol (final concentration) for maintenance of plasmids. Cultures were incubated overnight at 37°C with shaking (200 rpm—Thermo Scientific MaxQ 6000). Resultant overnight cultures were diluted (1:200) into flasks containing either 1 liter of LB media, supplemented with 3% glucose (w/v) and 34 μg/mL Chloramphenicol or 600 ml of autoclaved waste-media (22 mM KH_2_PO_4_, 22 mM Na_2_HPO_4_, 85 mM NaCl, 0.1% [v/v] NH_4_Cl, 2 mM MgSO_4_, 0.1 mM CaCl_2_, 0.4% glucose, 2% [w/v] mixed non-recyclable waste—80% cellulosic, 20% plastic and dH_2_0), supplemented with 34 μg/mL Chloramphenicol. Cultures were subsequently incubated for 24 hours or 48 hours at 37°C with shaking (200 rpm—Thermo Scientific MaxQ 6000)

### P(3HB) purification

This method is scaled down from an existing P(3HB) purification protocol [19]. Briefly, 1-liter production cultures were centrifuged at 4000 rpm (Beckman J2–M1) for 15 minutes. Waste-media cultures were filtered through Whatman filter paper in order to remove residual waste material before centrifugation. Post-centrifugation, bacterial cell pellets were re-suspended in phosphate-buffered saline (PBS), transferred into 50 ml tubes and centrifuged as described above. Cell pellets were washed with PBS and incubated for 30 minutes at room temperature in 1% (v/v in PBS) Triton-X 100. For the final purification of P(3HB), cells were centrifuged (4000 rpm—Beckman J2–M1 for 10 minutes), washed with PBS and incubated in aqueous sodium hypochlorite for 60 minutes at 30°C. The resultant purified P(3HB) granules were centrifuged (4000 rpm—Beckman J2–M1 for 10 minutes), washed with distilled water and dried overnight at 37°C. To determine the weight of the purified P(3HB), the weight of the 50 ml tube was subtracted from the combined weight of the 50 ml tube and P(3HB).

### Gene expression analysis with quantitative real-time PCR (qRT-PCR)

To analyse *phaCAB* gene expression we adapted a previously described qRT-PCR protocol [7]. Briefly, 1 ml of each *phaCAB*-engineered *E*. *coli* strain was harvested after 24 h of growth in 100 ml LB, supplemented with 3% glucose (w/v) and 34 μg/mL Chloramphenicol. To stabilise RNA, 400 μl of RNA Protect Bacteria reagent (Qiagen, Hilden, Germany) was added to each cell pellet. Cell pellets were stored at −80°C until RNA extraction. ZR Fungal/Bacteria RNA Microprep kits (Zymo Research, CA, USA, # R2010) were used to harvest total RNA. To eliminate any remaining plasmid or genomic DNA, the samples were treated with RNase-free DNase I (NEB, Beverly, MA, USA) for 10 minutes, during the RNA extraction process. RNA purity and concentration was determined using a Nanodrop ND-1000 spectrophotometer (Thermo Scientific). cDNA synthesis from 500ng of total RNA was performed using the iScript Select cDNA synthesis kit (Bio-Rad, Richmond, CA, USA).

cDNA templates were used to determine gene expression levels by quantitative real-time PCR. Primers for *phaA*, *phaB* and *phaC* were designed according to previously published sequences [7]. To perform real-time quantitative PCR, the iQ SYBR green Supermix kit (Bio-Rad) was used and the reactions were run on a Mastercycler ep. realplex (Eppendorf, Hamburg, Germany). The PCR reaction conditions were: 3 minutes at 95°C and then 40 cycles of 15 seconds at 95°C, 1 minute at 60°C. To confirm the specificity of the PCR primers, a melting curve analysis was carried out. Data were analysed via the relative standard curve method and subsequently normalised to native *phaCAB* samples. Data were analysed from three biological replicates.

### Flow cytometry analysis

Flow cytometry analysis of P(3HB) content was carried out as previously described [17,18,20]. Briefly, *E*. *coli* strain MG1655 carrying either empty vector, native *phaCAB*, constitutive *phaCAB* or hybrid *phaCAB* constructs were grown in 5 ml of waste-media (22 mM KH_2_PO_4_, 22 mM Na_2_HPO_4_, 85 mM NaCl, 0.1% [v/v] NH_4_Cl, 2 mM MgSO_4_, 0.1 mM CaCl_2_, 0.4% glucose, 2% [w/v] mixed non-recyclable waste—80% cellulosic, 20% plastic and dH_2_0), supplemented with 34 μg/mL Chloramphenicol. Cultures were subsequently incubated for 36 h at 37°C with shaking (200 rpm—Thermo Scientific MaxQ 6000). 1 ml of each overnight culture were centrifuged (8000 rpm—Eppendorf Minispin), washed with 1 ml PBS, and fixed with 35% Ethanol [v/v] at room temperature for 15 minutes. Post-fixation, cultures were centrifuged (8000 rpm—Eppendorf Minispin), re-suspended in 1ml PBS and stained with Nile Red (Sigma-Aldrich, MO, USA, #72485–100MG) to a final concentration of 20 μg/ml for 10 minutes on ice. Nile Red stained *E*. *coli* were diluted (1:100) into PBS and analyzed via flow cytometry. Around 65,000 cells per sample were loaded onto a BD-FACScan flow cytometer for detection of Nile Red staining (FL5, Ex 560, Em 610 nm) and data analysis from three biological replicates was carried out using FlowJo (vX 10.0.7r2) software. The background signal, as determined by the average geometric mean (FL-5) of Nile Red stained, empty vector transformed *E*. *coli*, was removed. Subsequently, these data were normalized to native-*phaCAB* engineered *E*. *coli*.

### Detection of 3HB from PhaZ1-depolymerized P(3HB) with the β-Hydroxybutyrate (Ketone Body) colorimetric assay kit

0.5 g of either purified P(3HB) or commercially sourced P(3HB) (Sigma-Aldrich, #363502–10G) were treated with 20μl PhaZ1 (0.36 μg/μl; BBa_K1149010) in 800 μl potassium phosphate buffer (100 mM pH 7.4), shaking at 200 rpm (Thermo Scientific MaxQ 6000) for 5 hours at 37°C. PhaZ1-treated and untreated samples were diluted 13% and 1.3% (v/v) in β-Hydroxybutyrate assay buffer (100 mM Tris-HCl pH 8.4) and analysed with the β-Hydroxybutyrate (Ketone Body) colorimetric assay kit (Cayman Chemical, MI, USA) to detect the presence of 3HB, as indicated by a yellow colour change.

## Supporting Information

S1 Supporting Information P(3HB) Modeling. This supplementary file includes a 31-page document that contains complete details about the P(3HB) synthesis model we constructed to forward-design the *phaCAB* operons that increase P(3HB) production.
(DOCX)

S2 Supporting Information P(3HB) Model. MATLAB Simbiology P(3HB) model files.
(ZIP)

S3 Supporting Information Nile Red staining of P(3HB). *E*. *coli* MG1655 transformed with either empty vector, native, constitutive or hybrid *phaCAB* constructs were cultured for 24 h at 37°C and 200 rpm shaking (Thermo Scientific MaxQ 6000) in 5 ml LB media, supplemented with 3% glucose (w/v) and 34 μg/mL Chloramphenicol. Liquid cultures were streaked onto LB-agar plates supplemented with 3% glucose (w/v), 34 μg/mL Chloramphenicol and 0.5 μg/ml Nile Red staining (Sigma-Aldrich, MO, USA). Plates were incubated for up to 48 h at 37°C and imaged with a Fuji Film LAS-5000 imager set to 473 nm excitation laser and Cy5 emission filter.
(PNG)

S4 Supporting Information Qualitative analysis of 3-hydroxybutyrate (3HB) from PhaZ1-depolymerized P(3HB). Purified P(3HB) from waste media cultured *E*. *coli* MG1655 carrying either empty vector, native *phaCAB*, constitutive *phaCAB* or hybrid *phaCAB* constructs were treated with or without the P(3HB) depolymerase, phaZ1. PhaZ1-treated and untreated samples were analysed with the β-Hydroxybutyrate (Ketone Body) colorimetric assay kit to detect the presence of 3HB, where a yellow colour change indicates the presence of 3HB.
(JPG)

S5 Supporting Information Engineered *phaCAB* operon sequences. (DOCX)

S6 Supporting Information *phaCAB* gene expression levels in engineered *E*. *coli*. *E*. *coli* MG1655 transformed with native [N], constitutive [C] or hybrid [H] *phaCAB* constructs were cultured in 5 ml LB media, supplemented with 3% glucose (w/v) for 24 h. Analysis of *phaCAB* gene expression was carried out using qRT-PCR and analyzed via the relative standard curve method. Experiments were carried out in triplicate and were normalized to native *phaCAB*–engineered *E*. *coli*. Error bars, +/- the standard deviation. Student t-test, *P<0.05 and **P <0.01.
(TIFF)
