# Supplementary material for: A Forward-Design Approach to Increase the Production of Poly-3-Hydroxybutyrate in Genetically Engineered Escherichia coli
Source: PLoS One. 2015 Feb 20;10(2):e0117202. doi: 10.1371/journal.pone.0117202 (PMC4336316; doi:10.1371/journal.pone.0117202)
Supplement: S1 Supporting Information — This supplementary file includes a 31-page document that contains complete details about the P(3HB) synthesis model we constructed to forward-design the phaCAB operons that increase P(3HB) production. (DOCX) [file pone.0117202.s001.docx]

**A forward-design approach to increase the production of poly-3-hydroxybutyrate in genetically engineered *Escherichia coli***

Supporting Information S1: P(3HB) Modeling

Table of Contents

1. Introduction 3

2. Model overview 5

3. Ordinary Differential Equations (ODE) 7

BDH2 (3-hydroxybutyrate dehydrogenase) 7

PhaB (Acetoacetyl-CoA reductase)and PhaC ((P(3HB) synthase)) 7

4. Enzyme kinetics 12

BDH2 (3-hydroxybutyrate dehydrogenase) 12

atoAD (Acetyl-CoA:acetoacetyl-CoA transferase (α and β subunits)) 14

phaB (Acetoacetyl-CoA reductase) 16

phaC (P(3HB) synthase) 18

atoB (acetyl-CoA acetyltransferase) 19

5. Simulation results 21

6. Metabolic considerations 24

Initial concentrations of metabolites 24

Table of initial and steady-state concentrations of the metabolites: 25

7. Model-guided design and optimization 26

Sensitivity analysis: species concentrations 26

Sensitivity analysis: enzyme concentrations 27

Scan with different levels of PhaB 28

Difference between promoter expressions after optimisation 29

References 31

# 1. Introduction

The model for our engineered system was constructed using Matlab Simbiology*^(^*[*^1^*](#_ENREF_1)*^)^*. Simbiology provides a graphical user interface (GUI) in which the poly-3-hydroxy-butyrate (P(3HB)) synthesis and gene regulation pathways can be visualised. This software was chosen as it contains solvers to ordinary differential equations that can be used to simulate the time evolution of the different biochemical reactions in our system. To construct the model in Simbiology, the different chemical species were linked together graphically in a block diagram formation provided by the GUI. Therefore, the relationships between each intermediate product and the enzymatic reactions that generate them could be described along the entire bioplastic synthesis pathway. The gene expression pathways were also constructed with transcription and translation taken into account. Then, the kinetic laws were specified for each link between the chemical species.

The main objective of the modelling was to compare how promoters of different strengths would affect the yield of P(3HB) in our engineered *E. coli*. To represent the different promoters that were tested, we utilised their different expression rates when specifying the kinetic law in the constitutive expression of PhaB and PhaC. With each expression rate, we simulated the model to obtain the time profile of P(3HB) synthesised. These results were then compared to determine which promoter would result in maximal P(3HB) production in the simulated time frame.

One point to note is that the synthesis pathway interacts with metabolites (NAD+ and NADPH etc.), which are themselves involved in many other reactions. Therefore, we adapted and extended upon an existing metabolic model by Dixon, 2011*^(^*[*^2^*](#_ENREF_2)*^)^*. This model (consisting of the TCA cycle and the glycolysis pathway) assisted in the estimation of different concentrations of metabolites available for our synthesis pathway to utilise. However, it should be noted, that when this metabolic model was coupled with our P(3HB) synthesis model, the concentrations of these metabolites were set at steady state levels. This is a rough estimation as the system responds dynamically to the environment and the steady state levels may change at different growth phases. Furthermore, there is competition between our synthesis pathway and the natural metabolism, which the model does not fully capture. To compensate for this, the ATP concentration over time was also simulated to give an indication of how the cells might react in response to the utilisation of several metabolites by our engineered *phaCAB* operon. Whilst the model could be further optimized, our model proved to be informative and were able to predict a general pattern that was experimentally verified, namely that use of the J23104 promoter did increase P(3HB) production in the constitutive and hybrid *phaCAB* operon variants. Possible refinements to the model could include further refinements to how the interactions between our engineered synthetic *phaCAB* operon and the natural metabolism of the cell are modelled.

**Key modeling outcomes**

- Scanning concentration of phaB and sensitivity analysis showed that increasing the concentration of phaB would increase the production rate of P(3HB) in our engineered *E. coli*.
- Simulations showed that the constitutive promoter J23104 could lead to a higher expression of pha B than the native promoter. As a result the wet lab team designed and constructed the bioparts BBa_K1149052 (Constitutive) and BBa_K1149051 (Hybrid), which significantly increased bioplastic production.
- Results from the metabolic model suggested that the synthesis of P(3HB) would be accompanied by a marked decrease in ATP over time and therefore this is informative in terms of the identification of any potential limitations of our engineered system when scaling for industrial implementation.

# 2. Model overview

The P(3HB) synthesis model was constructed and simulated using the Simbiology toolbox of Matlab. The model is comprised of the glycolysis pathway, the tricarboxylic acid (TCA) cycle and the *phaCAB* synthetic pathway. These models were coupled in order to reflect the metabolic flux of several metabolites (e.g. NAD+ and NADPH) between these pathways and thus their influence on P(3HB) production.


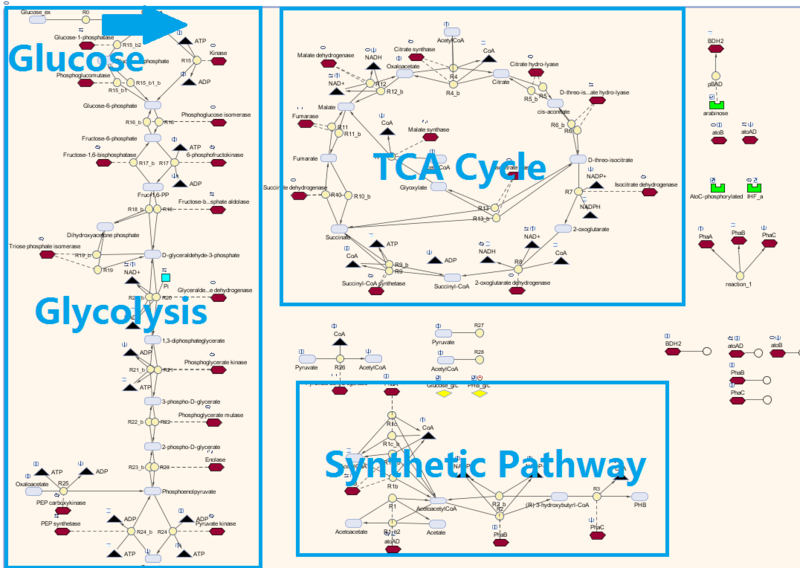
**Model overview.** This figure shows an overview of the model that was constructed to simulate P(3HB) production. The Glycolysis and TCA cycle models were adapted from Dixon, 2011*^(^*[*^2^*](#_ENREF_2)*^)^* and then coupled with different engineered *phaCAB* operon designs as part of our synthetic pathway.


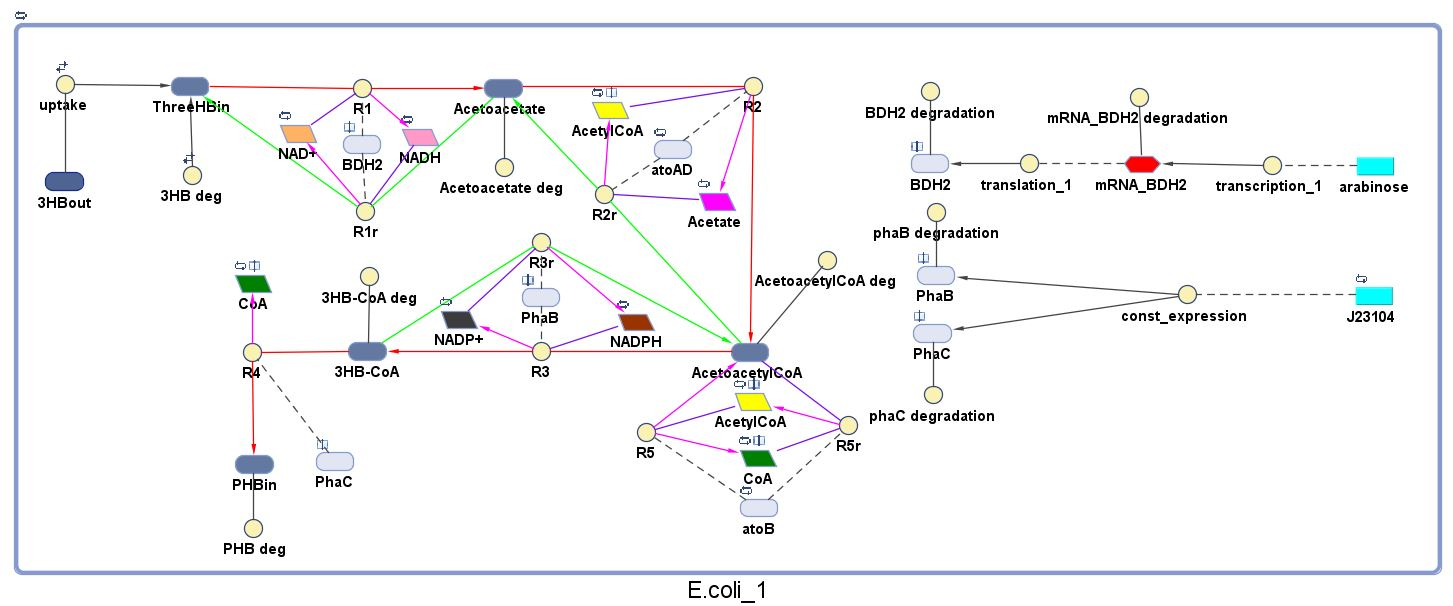

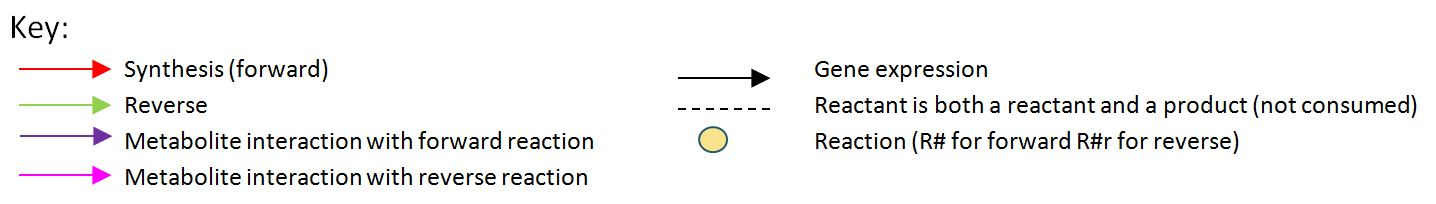
**Overview of the synthetic pathway model.** The pathways shown on the right are genetic expressions of the 3 enzymes involved in the plastic synthesis pathway, whereas the pathways on the left represent the actual P(3HB) production pathway. The synthetic pathway was primarily used for a sensitivity analysis by assuming minimum interference to the cell metabolism.

As can be seen in the Key (above), the yellow, solid circle represents a reaction object and for each object parameter the following needs to be specified for the simulation to work:

- Rate equation or kinetic law (e.g. mass action, Michaelis-Menten etc.)
- Parameters in the rate equation
- Species involved in the reaction
- Value and units for each parameter

Simbiology will then use an ODE solver (ode15 or sundial) to solve these ODEs and give a plot of the specified output(s) (concentration level of a species over time, for instance).

- Note that 3HB deg, Acetoacetate deg, AcetoacetylCoA deg and 3HB-CoA deg are degradation rates (all = 0.035/min, please see assumptions below) of the corresponding species formed along the P(3HB) synthesis pathway.

# 3. Ordinary Differential Equations (ODE)

**Genetic regulations and assumptions**

## BDH2 (3-hydroxybutyrate dehydrogenase)

**
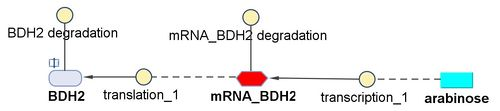
**

**
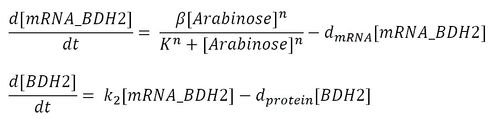
**Schematic of the gene expression of BDH2 as seen in the model diagram. ODE shown below the pathway.

## PhaB (Acetoacetyl-CoA reductase)and PhaC ((P(3HB) synthase))

**
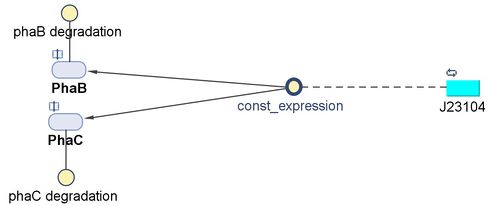
**

**
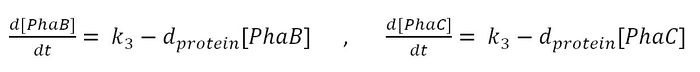
**

Schematic of the gene expression of PhaB and PhaC as shown in the model diagram. ODE shown below the pathway.

**Values sources and assumptions**

| **Parameter** | **Description** | **Value** | **Units** | **Sources** | **Assumptions/Notes** |
| --- | --- | --- | --- | --- | --- |
| **β** | Max rate of transcription | 0.032 | mM/min | Please see derivation 1 below. | Please see derivation 1 below. |
| **n** | Hill coefficient | 2.0 | dimensionless | <http://parts.igem.org/Part:pSB1C3?title=Part:pSB1C3> | For pBAD strong. Taken from the parts registry page. Rounded to 2.0 from 2.26 as Simbiology wouldn't allow a non-integer value for such parameter. |
| **K** | Activation coefficient | 0.0031 | mM | <http://parts.igem.org/Part:BBa_K206000:Characterization> | For pBAD strong. Taking the "switch point" (from the corresponding parts registry page) as the activation coefficient. |
| **d_mRNA_** | mRNA degradation rate | 0.035 | 1/min | *^(^*[*^3^*](#_ENREF_3)*^)^* | There is no active degradation pathway and that dilution is the dominant way by which it degrades. Rate = ln2/doubling time, where doubling time of strain MG1655 = 20min. Assuming steady-state growth in LB broth as presented in paper. rate = ln2/20 = 0.035/min |
| **d_protein_** | Protein degradation rate | 0.035 | 1/min | *^(^*[*^3^*](#_ENREF_3)*^)^* | There is no active degradation pathway and that dilution is the dominant way by which it degrades. Rate = ln2/doubling time, where doubling time of strain MG1655 = 20min. Assuming steady-state growth in LB broth as presented in paper. rate = ln2/20 = 0.035/min |
| **k_2_** | Protein production rate (BDH2) | 4.7 | 1/min | Please see derivation 2 below. | Please see derivation 2 below. |
| **k_3_** | Protein production rate (PhaCB) | 0.58 | mM/min | Please see derivation 3 below. | Please see derivation 3 below. |
| **[Arabinose]** | Concentration of arabinose | Initial: 0.008 | mM | See section 6. "Initial concentrations of metabolites" |  |
| **[mRNA]** | Concentration of mRNA | - | mM | - | - |
| **[BDH2]** | Concentration of BDH2 | - | mM | - | - |
| **[PhaB]** | Concentration of PhaB | - | mM | - | - |
| **[PhaC]** | Concentration of PhaC | - | mM | - | - |

**Derivations**

**1.Derivation of the maximal expression rate,β**

- Average molecular weight (Mw) of a base pair = 660g/mol *^(^*[*^4^*](#_ENREF_4)*^,^* [*^5^*](#_ENREF_5)*^)^*
- Average mass of a base pair = 660g/mol x 1.66x10^-24^ = 1.1x10^-21^g
- Volume of an *E.coli* cell = 1µm^3^ = 1x10^-15^L *^(^*[*^6^*](#_ENREF_6)*^)^*
  - Mass concentration = 
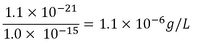

  - Molar concentration of 1 base pair in the volume of *E.coli* = 
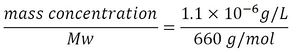
 = 1.66x10^-6^ mM
- BioBrick assembly plasmid pSB1C3 is a high copy number plasmid (100-300 copies per cell) [(http://parts.igem.org/Part:pSB1C3?title=Part:pSB1C3)](http://parts.igem.org/Part:pSB1C3?title=Part:pSB1C3)
  - Assume 200 copies per cell
- Concentration of the gene per cell = N x 200 x 1.66x10^-6^mM, where N = number of base pairs
  - Concentration of the gene BDH2 (N = 768) in the volume of an *E.coli* cell is = 0.25mM
- Transcription rate*^(^*[*^6^*](#_ENREF_6)*^)^* in *E.coli*= 80bp/s = 80 x 1.66x10^-6^mM/s = 80 x 1.66x10^-6^ x 60mM/min = 7.97x10^-3^mM/min
- Rate of mRNA_BDH2 production under the control of pBAD = 7.97x10^-3^ ÷ 0.25 = **0.032/min**

**2.Protein production rate of BDH2, k_2_**

- Average molecular weight(Mw) of an amino acid(aa)= 110g/mol *^(^*[*^7^*](#_ENREF_7)*^,^* [*^8^*](#_ENREF_8)*^)^*
- Average mass of an amino acid = 110g/mol x 1.66x10^-24^=1.83x10^-22^g/L
  - Mass concentration of one aa in the volume of an *E.coli* = 
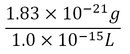
 = 1.83x10^-6^g/L
  - Molar concentration of one aa = 
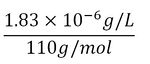
 = 1.66x10^-5^mM
- Translation rate = 20aa/s = (20 x 1.66x10^-5^ x 60)mM/min = 0.020mM/min
- BDH2 comprises of 256aa *^(^*[*^9^*](#_ENREF_9)*^)^*
  - Concentration of BDH2's aa in the volume of an *E.coli*= 1.66x10^-5^mM x 256 = 4.25x10^-3^mM
- Rate of protein production = 0.020 ÷ 4.25x10^-3^ = **4.7/min**

**3.Protein production rate for PhaB and PhaC, k_3_**

- Relative promoter strengths: J23104 = 1.3RPU, J23101 = 1.0RPU. *^(^*[*^10^*](#_ENREF_10)*^)^*
  - 104 is 1.3x stronger than 101.
- In absolute units: take GFP synthesis rate (molecules per min per cell) and approximate that as a generic protein synthesis rate for the promoter.
  - GFP synthesis rate of 101 = 2232 molecules per min per cell. *^(^*[*^10^*](#_ENREF_10)*^)^*
  - GFP synthesis rate of 104 = 1.3 x 2232 = 2902 molecules per min per cell
- Assume 1 molecule in an *E.coli* cell gives a concentration of 1nM.
  - GFP synthesis rate of 104 = 2902 x 1nM = 2.9x10^-6^nM/min per cell
- Plasmid copy number assumed as 200 (as in derivation 1)
  - GFP synthesis rate of 104 in our *E.coli* = 200 x 2.9x10^-6^ = 0.00058nM/min = **0.58mM/min**

# 4. Enzyme kinetics

- - v = velocity of reaction
  - Ac-CoA = acetyl-CoA
  - AcAc-CoA = Acetoacetyl-CoA
  - AcAc = acetoacetate

## BDH2 (3-hydroxybutyrate dehydrogenase)

**Reaction R1:** 
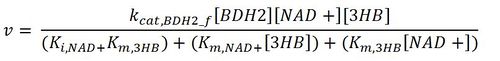


**Reaction R1r:** 
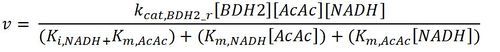


##### **Values, sources and assumptions**

| **Parameter** | **Description** | **Value** | **Units** | **Source** | **Assumptions/Notes** |
| --- | --- | --- | --- | --- | --- |
| **k_cat,BDH2_f_** | Turnover number of BDH2 in forward reaction | 22200 | 1/min | *^(^*[*^11^*](#_ENREF_11)*^)^* | Sequences derived from *Pseudomonas fragi* but kinetic values from expression and purification of enzymes in *E. coli* XL1 Blue |
| **k_cat,BDH2_r_** | Turnover number of BDH2 in reverse reaction | 7200 | 1/min | *^(^*[*^11^*](#_ENREF_11)*^)^* | Sequences derived from *Pseudomonas fragi* but kinetic values from expression and purification of enzymes in *E. coli* XL1 Blue |
| **K_i,NAD+_** | Inhibition constant of BDH2 with NAD+ | 2.5 | mM | *^(^*[*^12^*](#_ENREF_12)*^)^* | Organism Tetrahymena pyriformis |
| **K_i,NADH_** | Inhibition constant of BDH2 with NADH | 1.1 | mM | *^(^*[*^12^*](#_ENREF_12)*^)^* | Organism Tetrahymena pyriformis |
| **K_m,AcAc_** | Michaelis constant for AcAc | 0.37 | mM | *^(^*[*^11^*](#_ENREF_11)*^)^* | Values in paper reference |
| **K_m,NAD+_** | Michaelis constant for NAD+ | 0.24 | mM | *^(^*[*^11^*](#_ENREF_11)*^)^* | Values in paper reference |
| **K_m,NADH_** | Michaelis constant for NADH | 0.010 | mM | *^(^*[*^11^*](#_ENREF_11)*^)^* | Values in paper reference |
| **K_m,3HB_** | Michaelis constant for 3HB | 0.80 | mM | *^(^*[*^11^*](#_ENREF_11)*^)^* | source organism *Pseudomonas fragi* |
| **[BDH2]** | Concentration of BDH2 | - | mM | - | - |
| **[NAD+]** | Concentration of NAD+ | initial: 1.6x10^-13^ | mM | See section 6. "Initial concentrations of metabolites" |  |
| **[3HB]** | Intracellular concentration of 3HB | 6.4x10^-14^ | mM | - | External concentration of 3HB assumed to be 0.01g. This is shared by 1.5x10^12^cells. Therefore, one cell has 0.01/1.5x10^12^ = 6.7x10^-15^ g. To convert it into mol/L: mass of 3HB/molar mass of 3HB = 6.7x10^-15^/104.1 = 6.4x10^-17^ M = 6.4x10^-14^ mM |
| **[AcAc]** | Concentration of AcAc | initial: 1.0x10^-13^ | mM | See section 6. "Initial concentrations of metabolites" |  |
| **[NADH]** | Concentration of NADH | initial: 2.5x10^-14^ | mM | See section 6. "Initial concentrations of metabolites" |  |

## atoAD (Acetyl-CoA:acetoacetyl-CoA transferase (α and β subunits))

**Reaction R2:** 
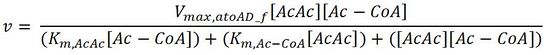


**Reaction R2r:** 
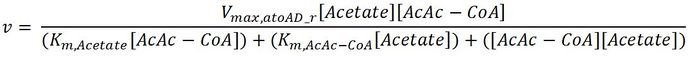


##### **Values, sources and assumptions**

| **Parameter** | **Description** | **Value** | **Units** | **Sources** | **Assumptions/Notes** |
| --- | --- | --- | --- | --- | --- |
| **V_max,atoAD_f_** | Maximum rate of atoAD in forward reaction | 0.00244 | mM/min | *^(^*[*^13^*](#_ENREF_13)*^)^* | Enzyme is naturally expressed in *E.coli*: assume the enzyme is stable during the time course of the simulation. |
| **V_max,atoAD_r_** | Maximum rate of atoAD in reverse reaction | 0.0108 | mM/min | *^(^*[*^13^*](#_ENREF_13)*^)^* | Enzyme is naturally expressed in *E.coli*: assume the enzyme is stable during the time course of the simulation. |
| **K_m,AcAc_** | Michaelis constant for AcAc | 1.86 | mM | *^(^*[*^13^*](#_ENREF_13)*^)^* | Enzyme is naturally expressed in *E.coli*: assume the enzyme is stable during the time course of the simulation. |
| **K_m,Ac-CoA_** | Michaelis constant for Ac-CoA | 0.26 | mM | *^(^*[*^14^*](#_ENREF_14)*^)^* | Enzyme is naturally expressed in *E.coli*: assume the enzyme is stable during the time course of the simulation. |
| **K_m,Acetate_** | Michaelis constant for Acetate | 53.1 | mM | *^(^*[*^15^*](#_ENREF_15)*^)^* | Enzyme is naturally expressed in *E.coli*: assume the enzyme is stable during the time course of the simulation. |
| **K_m,AcAc-CoA_** | Michaelis constant for AcAc-CoA | 0.035 | mM | *^(^*[*^13^*](#_ENREF_13)*^)^* | Enzyme is naturally expressed in *E.coli*: assume the enzyme is stable during the time course of the simulation. |
| **[AcAc]** | Concentration of AcAc | initial: 1.0x10^-13^ | mM | See section 6. "Initial concentrations of metabolites" |  |
| **[Acetate]** | Concentration of Acetate | initial: 1.0x10^-13^ | mM | See section 6. "Initial concentrations of metabolites" |  |
| **[AcAc-CoA]** | Concentration of AcAc-CoA | initial: 1.0x10^-13^ | mM | See section 6. "Initial concentrations of metabolites" |  |
| **[Ac-CoA]** | Concentration of Ac-CoA | initial: 1.0x10^-14^ | mM | See section 6. "Initial concentrations of metabolites" |  |

## phaB (Acetoacetyl-CoA reductase)

**Reaction R3:** 
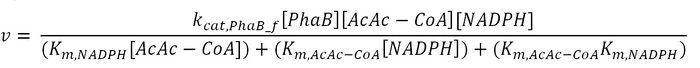


**Reaction R3r:**
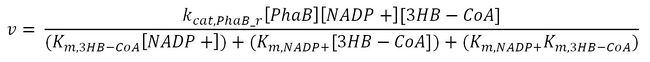


##### **Values, sources and assumptions**

| **Parameter** | **Description** | **Value** | **Units** | **Sources** | **Assumptions/Notes** |
| --- | --- | --- | --- | --- | --- |
| **k_cat,PhaB_f_** | Turnover number of PhaB in forward reaction | 6120 | 1/min | *^(^*[*^16^*](#_ENREF_16)*^)^* | Gene in Ralstonia eutropha was engineered to put in E.coli and then purified |
| **k_cat,PhaB_r_** | Turnover number of PhaB in reverse reaction | 3600 | 1/min | *^(^*[*^17^*](#_ENREF_17)*^)^* | originally in Zoogloea ramigera, expressed in E.coli and then purified |
| **K_m,NADPH_** | Michaelis constant for NADPH | 0.15 | mM | *^(^*[*^16^*](#_ENREF_16)*^)^* | Gene in Ralstonia eutropha was engineered to put in E.coli and then purified |
| **K_m,AcAc-CoA_** | Michaelis constant for AcAc-CoA | 0.0057 | mM | *^(^*[*^16^*](#_ENREF_16)*^)^* | Gene in Ralstonia eutropha was engineered to put in E.coli and then purified |
| **K_m,NADP+_** | Michaelis constant for NADP+ | 0.0060 | mM | *^(^*[*^18^*](#_ENREF_18)*^)^* | purified from Methylobacterium extorquens |
| **K_m,3HB-CoA_** | Michaelis constant for 3HB-CoA | 0.026 | mM | *^(^*[*^17^*](#_ENREF_17)*^)^* | originally in Zoogloea ramigera, expressed in E.coli and then purified |
| **[PhaB]** | Concentration of PhaB | - | mM | - | - |
| **[AcAc-CoA]** | Concentration of AcAc-CoA | initial:1.0x10^13^ | mM | See section 6. "Initial concentrations of metabolites" |  |
| **[NADPH]** | Concentration of NADPH | initial:3.8x10^14^ | mM | See section 6. "Initial concentrations of metabolites" |  |
| **[NADP+]** | Concentration of NADP+ | initial:1.5x10^13^ | mM | See section 6. "Initial concentrations of metabolites" |  |
| **[3HB-CoA]** | Concentration of 3HB-CoA | - | mM | - | - |

## phaC (P(3HB) synthase)

**Reaction R4:** 
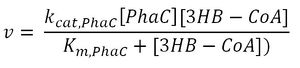


##### **Values, sources and assumptions**

| **Parameter** | **Description** | **Value** | **Units** | **Sources** | **Assumptions/Notes** |
| --- | --- | --- | --- | --- | --- |
| **k_cat,PhaC_** | Turnover number of PhaC | 1680 | 1/min | [[52]](http://ac.els-cdn.com/S0003986101925226/1-s2.0-S0003986101925226-) | - |
| **K_m,PhaC_** | Michaelis constant of PhaC with 3HB-CoA | 0.14 | mM | [[53]](http://www.brenda-enzymes.org/php/result_flat.php4?ecno=2.3.1.B5) | Enzyme is naturally expressed in *E.coli*: assume the enzyme is stable during the time course of the simulation. |
| **[PhaC]** | Concentration of PhaC | - | mM | - | - |
| **[3HB-CoA]** | Concentration of 3HB-CoA | - | mM | - | - |

## atoB (acetyl-CoA acetyltransferase)

**Reaction R5:** 
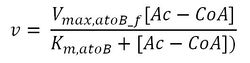


**Reaction R5r:**
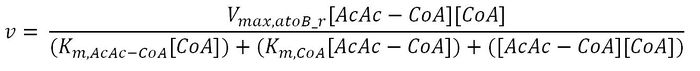


##### Values, sources and assumptions

| **Parameter** | **Description** | **Value** | **Units** | **Sources** | **Assumptions/Notes** |
| --- | --- | --- | --- | --- | --- |
| **V_max,atoB_f_** | Maximum rate of atoB in forward reaction | 3.8x10^-5^ | mM/min | [[54]](http://www.ncbi.nlm.nih.gov/pubmed/9904) | Enzyme is naturally expressed in *E.coli*: assume the enzyme is stable during the time course of the simulation. |
| **V_max,atoB_r_** | Maximum rate of atoB in reverse reaction | 8.5x10^-4^ | mM/min | [[55]](http://www.ncbi.nlm.nih.gov/pubmed/9904) | Enzyme is naturally expressed in *E.coli*: assume the enzyme is stable during the time course of the simulation. |
| **K_m,atoB_** | Michaelis constant of atoB with Ac-CoA | 0.47 | mM | [[56]](http://www.sciencedirect.com/science/article/pii/0003986176901521) | Enzyme is naturally expressed in *E.coli*: assume the enzyme is stable during the time course of the simulation. |
| **K_m,AcAc-CoA_** | Michaelis constant for AcAc-CoA | 0.1 | mM | [[57]](http://www.sciencedirect.com/science/article/pii/0003986176901521) | Enzyme is naturally expressed in *E.coli*: assume the enzyme is stable during the time course of the simulation. |
| **K_m,CoA_** | Michaelis constant for CoA | 0.25 | mM | [[58]](http://www.sciencedirect.com/science/article/pii/0003986176901521) | Enzyme is naturally expressed in *E.coli*: assume the enzyme is stable during the time course of the simulation. |
| **[Ac-CoA]** | Concentration of Ac-CoA | initial: 1.0x10^-13^ | mM | See section 6. "Initial concentrations of metabolites" |  |
| **[AcAc-CoA]** | Concentration of AcAc-CoA | initial: 1.0x10^-13^ | mM | See section 6. "Initial concentrations of metabolites" |  |
| **[CoA]** | Concentration of CoA | initial: 5.5x10^-13^ | mM | See section 6. "Initial concentrations of metabolites" |  |

**Reaction kinetics and mechanisms**

List of enzymes and the corresponding mechanisms by which they work in the synthesis pathway

- BDH2: Ordered-sequential bi-bi mechanism*^(^*[*^19^*](#_ENREF_19)*^)^*
- atoAD: Ping-pong bi-bi mechanism *^(^*[*^13^*](#_ENREF_13)*^)^*
- atoB: Ping-pong bi-bi mechanism *^(^*[*^13^*](#_ENREF_13)*^)^*
- PhaB: Ordered-sequential bi-bi mechanism ^(2)^
- PhaC: Michaelis-Menten *^(^*[*^20^*](#_ENREF_20)*^)^*

# 5. Simulation results

Using the values presented in the tables above, the deterministic model was simulated to see how much P(3HB) could be formed inside the cell. However, with the single cell model, it would be difficult to reflect how much of the plastic could actually be formed on a macroscopic scale. Therefore, modifications to the simulation algorithm were needed to give scaled-up simulation results.

In the scaled-up regime, though, new limitations were encountered. For example, growth, cell division and the concentration gradient that each cell encounters and how much can be taken up by the cells. Given the data available, what could be done was to assume that the bacteria were in a bioreactor, with constant nutrient and oxygen supply, and that they were in the phase of stationary growth (please see assumptions and explanations under the graphs below).

Here, three graphs are presented to show P(3HB)formation over time. The first two graphs are (i)single-cell model results, concentration in mM and (ii) single-cell model results, concentration in g/L. The first graph was the original graph produced from Simbiology as all concentrations were expressed in mM in the model. However, to gain a more intuitive sense of how much could be produced g/L was used. The third graph is the scaled-up simulation result.

**(i) Please see assumptions and explanations below**


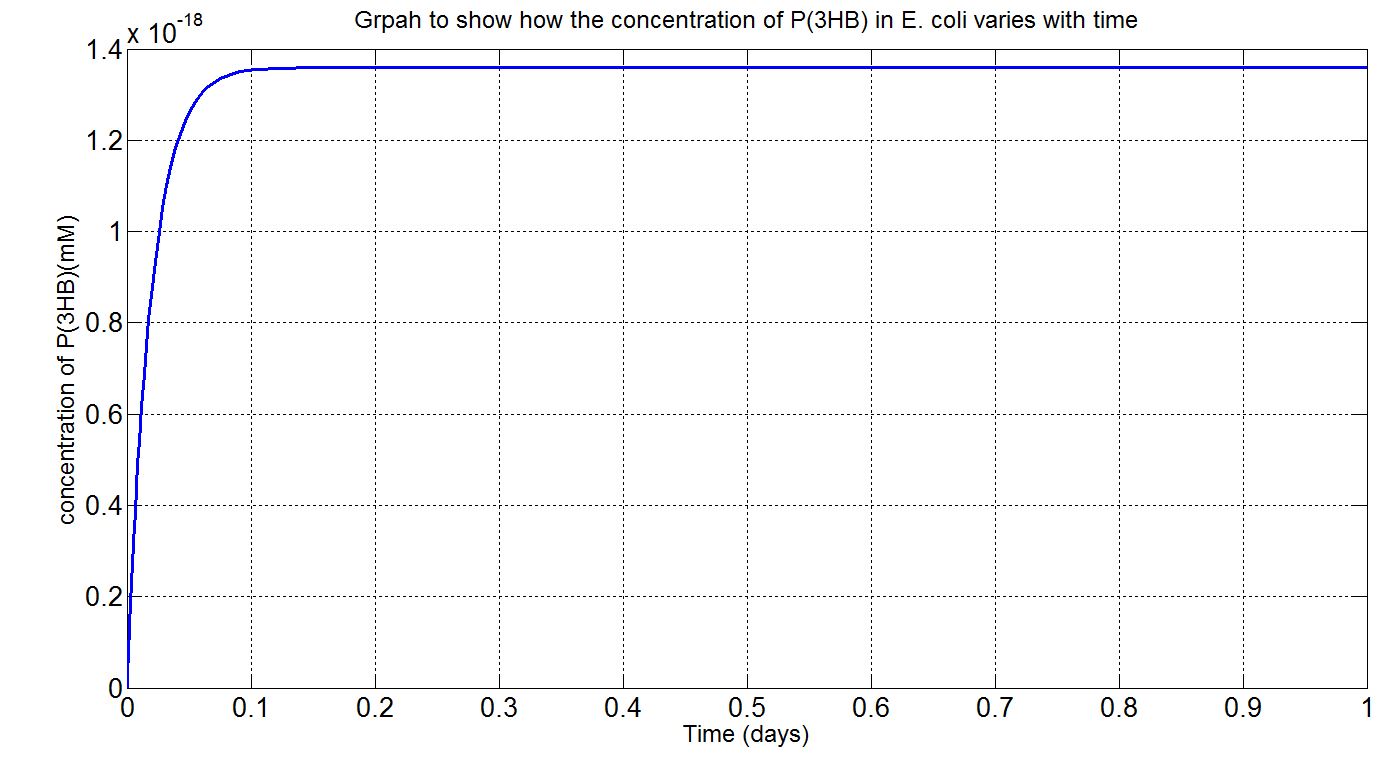


**(ii) please see assumptions and explanations below**


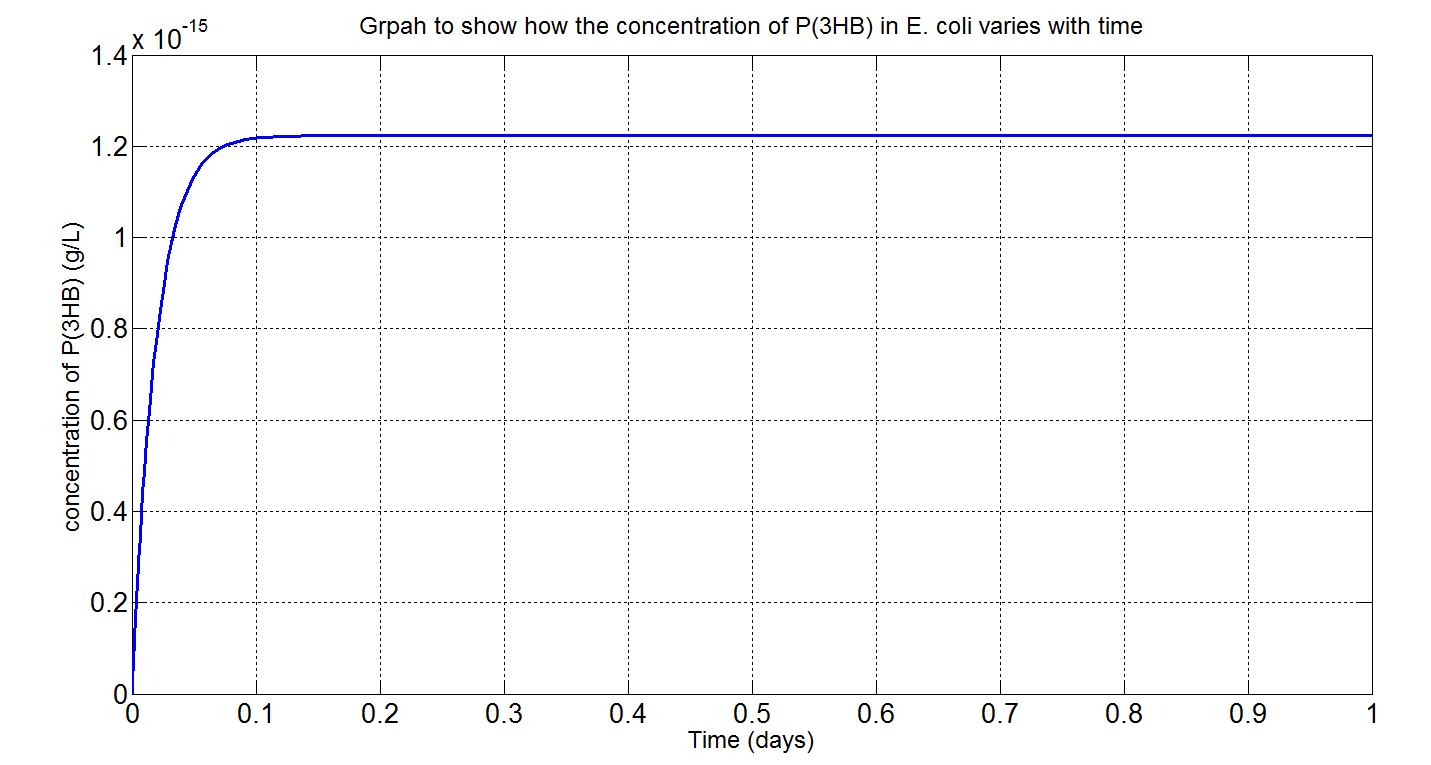


**(iii) please see assumptions and explanations below**


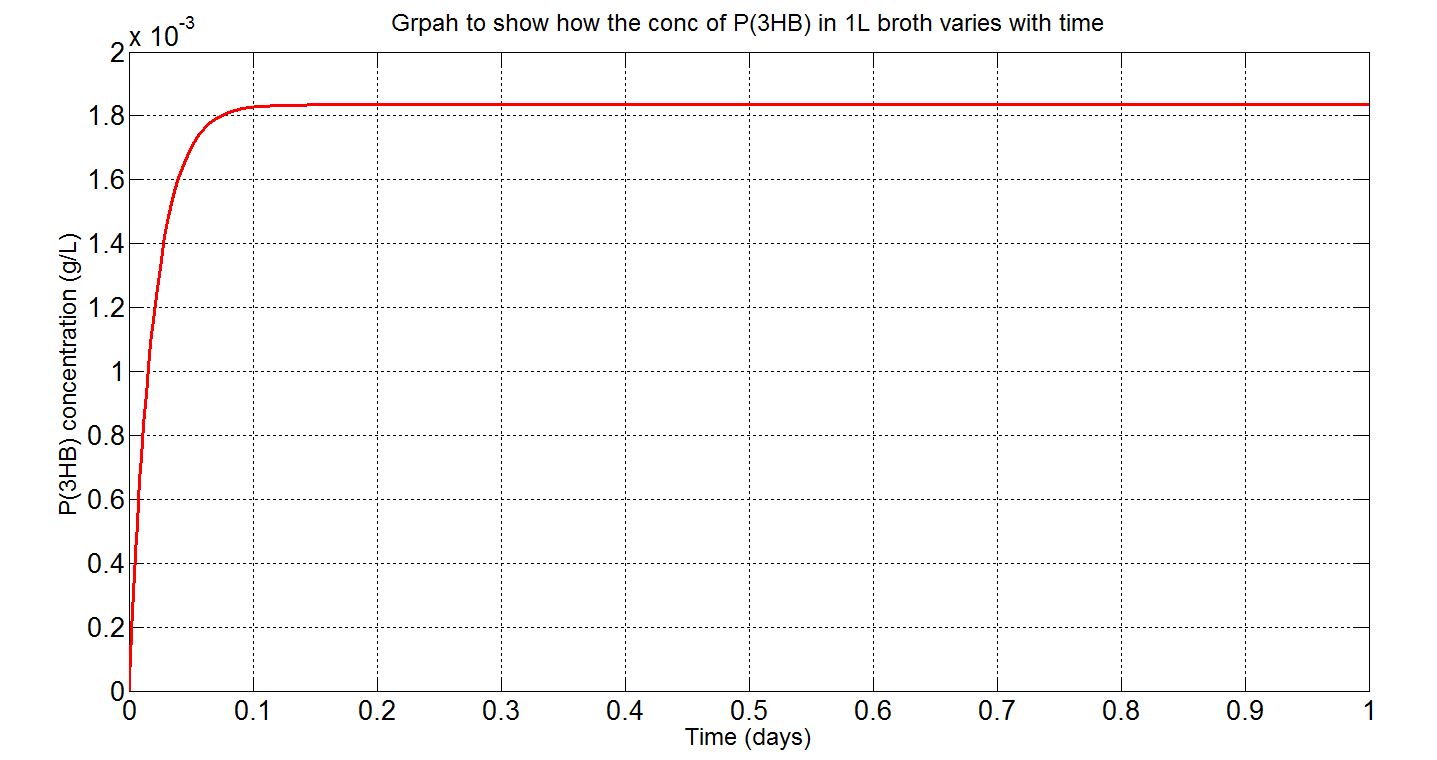


**Graphs (i) and (ii): assumptions and explanations**

- To convert from mM to g/L:
  - Molecular weight(Mw) of P(3HB) = 1.8x10^12^g/mol *^(^*[*^21^*](#_ENREF_21)*^)^*

(Please note that this value was taken from a paper about the "Production of poly(3-hydroxybutyrate-co-4-hydroxybutyrate) in recombinant Escherichia coli grown on glucose" instead of the production of poly(3-hydroxybutyrate). However, this is the closest we managed to get as far as species, substrate and type of plastic are concerned.

Also, it was noticed that the molecular weight would change depending on the cultivation time (as conditions such as growth and pH can also affect it) and it was hard to find a paper that contains the exact same conditions and the range of cultivation times for our simulations. Furthermore, in reality there wouldn't be a single value of molecular weight obtained, but a range of values (known as polydispersity)*^(^*[*^22^*](#_ENREF_22)*^)^*. Therefore, it was assumed that the molecular weight would remain constant during the time course of the simulation and that the value should be considered as an average.)

- P(3HB)concentration in mM x 10^-3^ x 1.8x10^12^g/mol = concentration in g/L

**Graph (iii): assumptions and explanations**

- Assume 1.5x10^12^cells in a 1L broth, calculated from value obtained from BioNumbers. A range was given: 1-2x10^9^ cells/ml, so 1.5x10^9^was taken. *^(^*[*^6^*](#_ENREF_6)*^)^*
  - Conditions and considerations accompanying this value are:
    - growth medium: LB broth
    - Cultivation temperature: 37°C
    - wild-type E. coli K-12 strain MG1655 (NB: though not exactly our engineered one, it is the correct strain)
- Assume stationary growth phase.
- Assume that the amount of P(3HB) accumulated during the time course of simulation doesn't exceed the cell's capacity or ability to contain it.
- Same metabolic considerations as above (single-cell results)
- Simbiology single-cell simulation results multiplied by the number of cells stated above to give the red curve.

# 6. Metabolic considerations

Our synthetic pathway involves several key metabolites from the pathway such as NADPH, NADP+, NADH and NAD+. The initial concentration of those metabolites can be determined from the metabolic model by assuming they are in their steady states.

## Initial concentrations of metabolites

The metabolic model for determining steady state concentrations is shown as below:


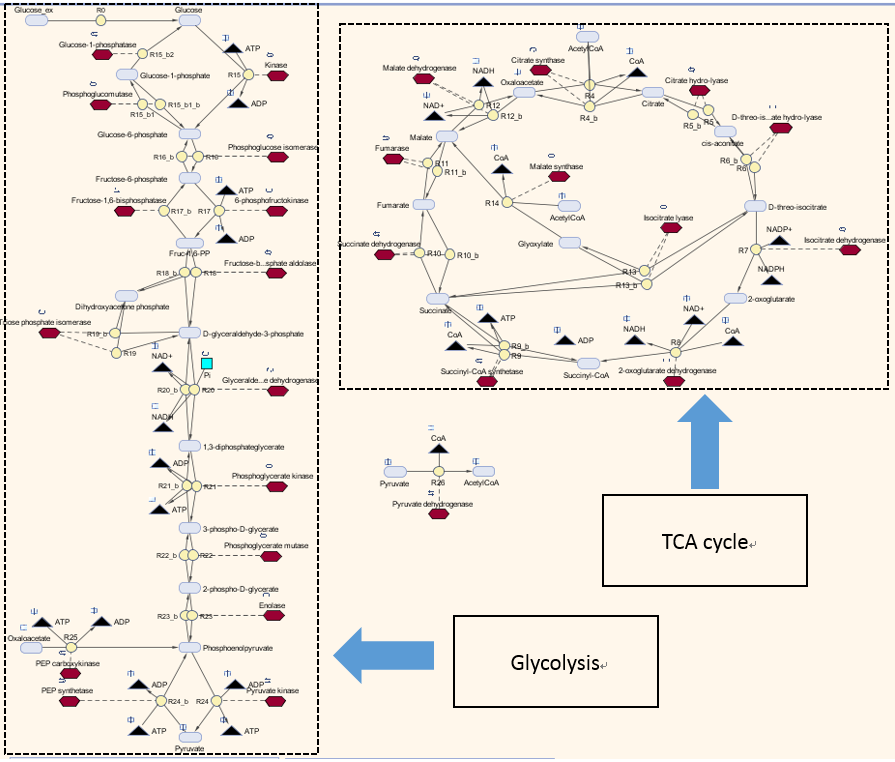


**Keys:**


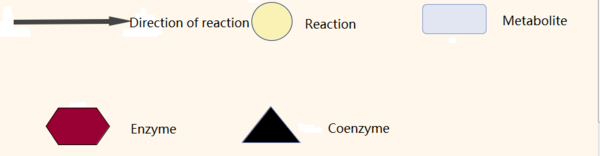


The initial concentrations of all metabolites and kinetic data are referenced in Dixon, 2011*^(^*[*^2^*](#_ENREF_2)*^)^*.

The simulation of the metabolites is:


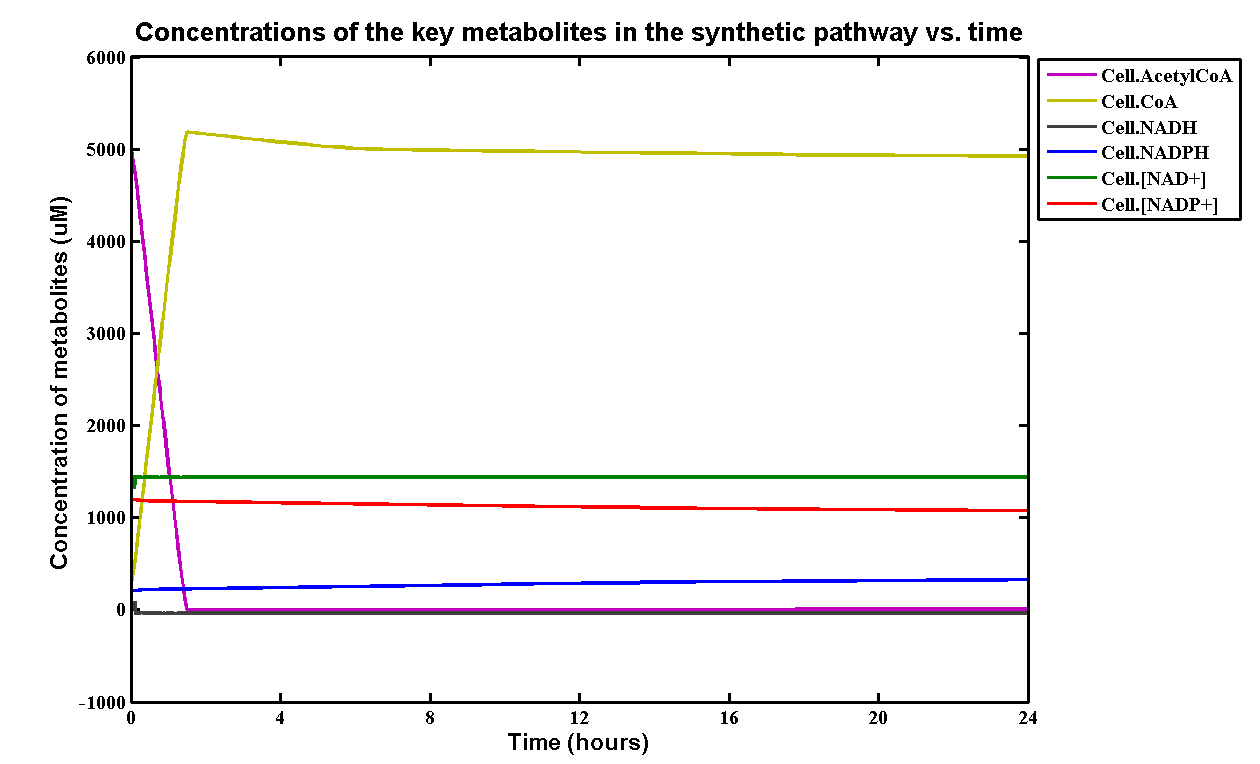


### Table of initial and steady-state concentrations of the metabolites:

| **Substrates** | **Initial concentration** | **Steady state concentration** | **Units** | **^Sources^** |
| --- | --- | --- | --- | --- |
| **NADH** | 200 | 250 | uM | *^(^*[*^2^*](#_ENREF_2)*^)^* |
| **NAD+** | 1200 | 1600 | uM | *^(^*[*^2^*](#_ENREF_2)*^)^* |
| **Acetyl-coA** | 1000 | 100 | uM | *^(^*[*^2^*](#_ENREF_2)*^)^* |
| **Coenzyme A** | 250 | 5500 | uM | *^(^*[*^2^*](#_ENREF_2)*^)^* |
| **NADPH** | 200 | 380 | uM | *^(^*[*^2^*](#_ENREF_2)*^)^* |
| **NADP+** | 1200 | 1100 | uM | ^(2)^ |

It should also be noted that acetate, acetoacetate and acetoacetyl-CoA cannot be predicted by the metabolic model. Therefore, their initial concentrations are rough estimates (value similar to other metabolites) such that a reasonable P(3HB) output can be produced.

# 7. Model-guided design and optimization

## Sensitivity analysis: species concentrations

In order to look at how we could increase the production of P(3HB) we decided to run a sensitivity analysis (also in Simbiology) to identify what species in the model P(3HB) is sensitive to, given the specific conditions with which we set the model. In other words, we calculated the time-dependent sensitivity of P(3HB) with respect to the initial conditions of other species (species formed along the synthesis pathway and enzymes involved)h*^(^*[*^23^*](#_ENREF_23)*^)^*

For a species x, whose concentration depends on time, it can be expressed as x(t). To calculate the sensitivity of x(t) with respect to another species y(t), the sensitivity with normalisation relative to the numerator x(t) can be calculated as:


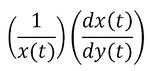


Therefore, the underlying algorithm for this sensitivity analysis of P(3HB) is:


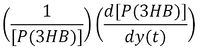


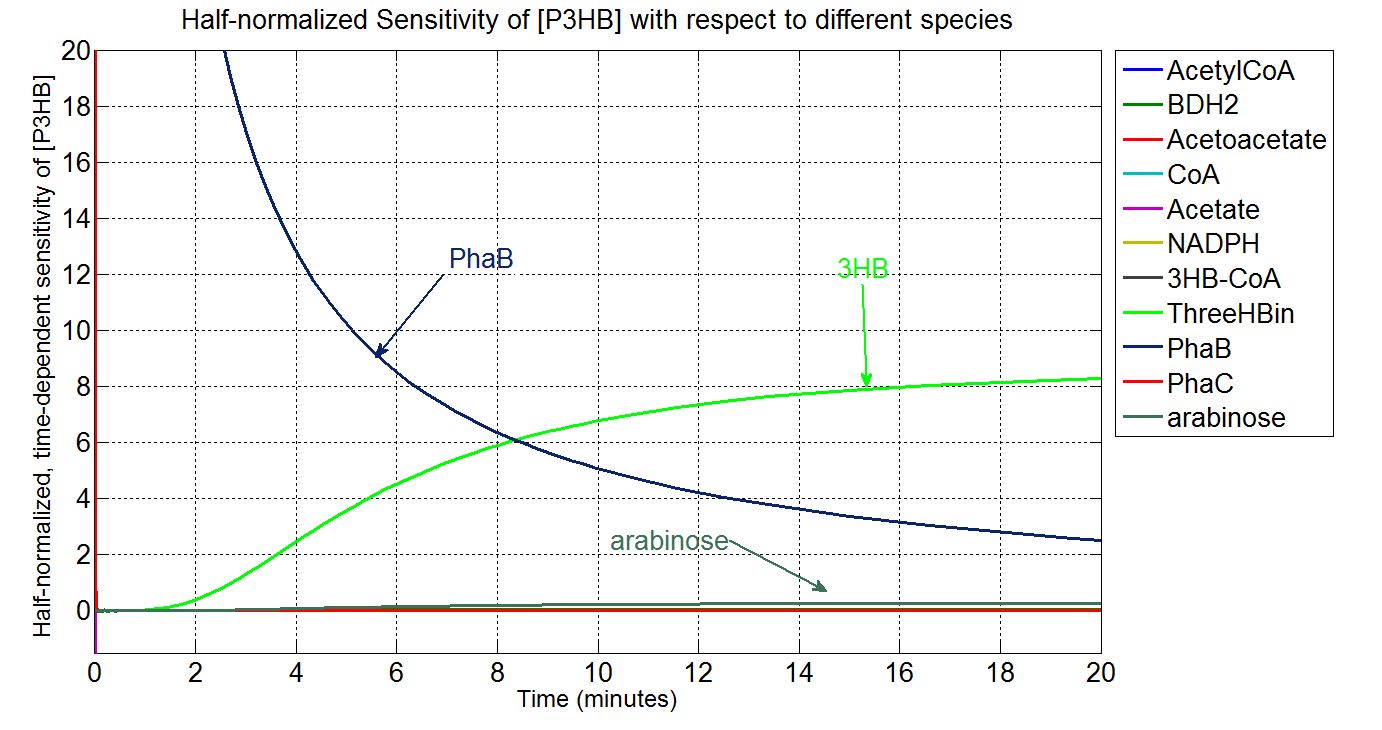


## Sensitivity analysis: enzyme concentrations

The sensitivity analysis of the enzymes involved in the synthetic pathway is carried out, which determine the most sensitive enzyme in the pathway

We plot a time integral of the sensitivity analysis which has the algorithm as:


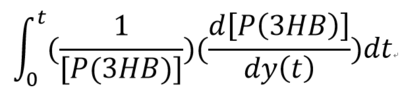


The sensitivity result is:


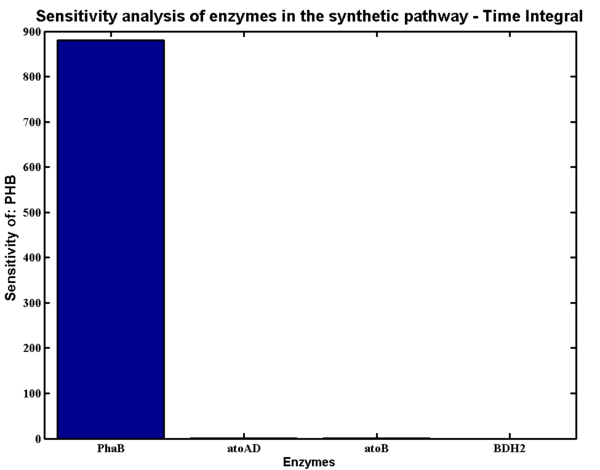


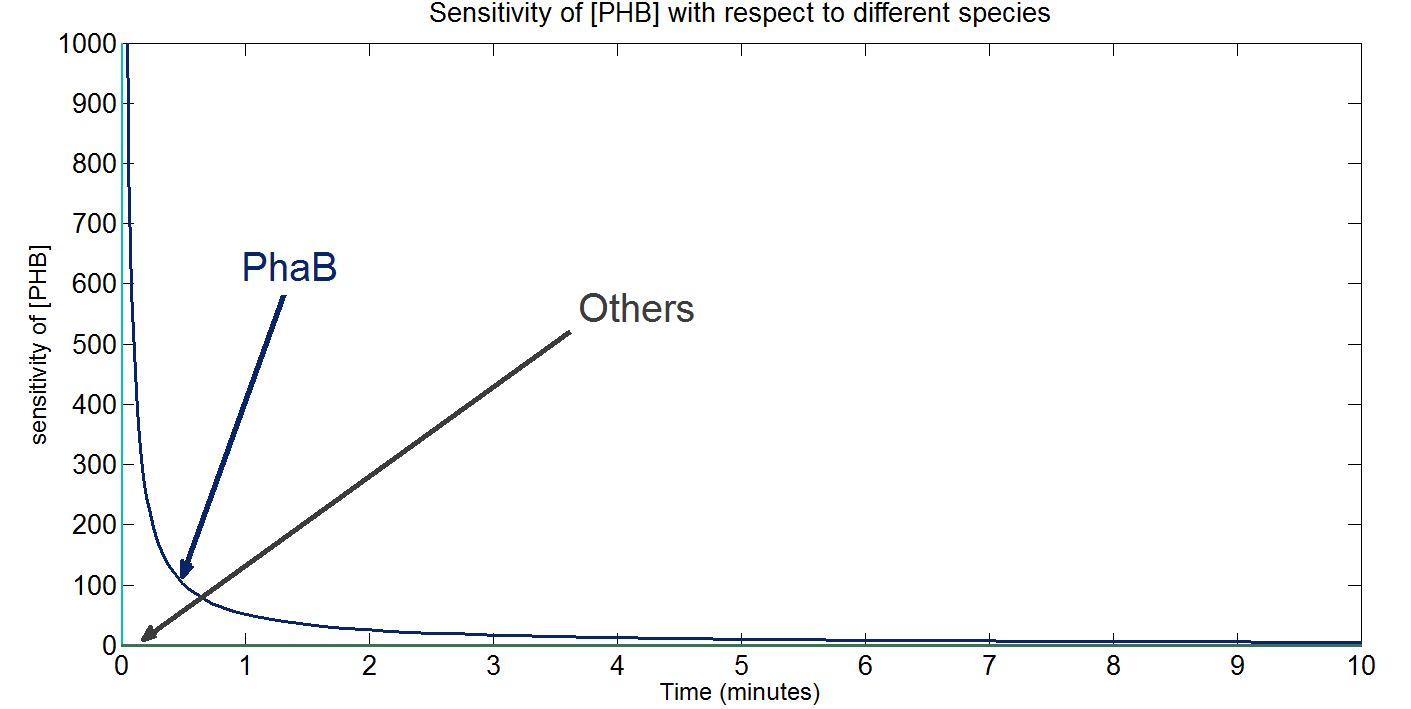

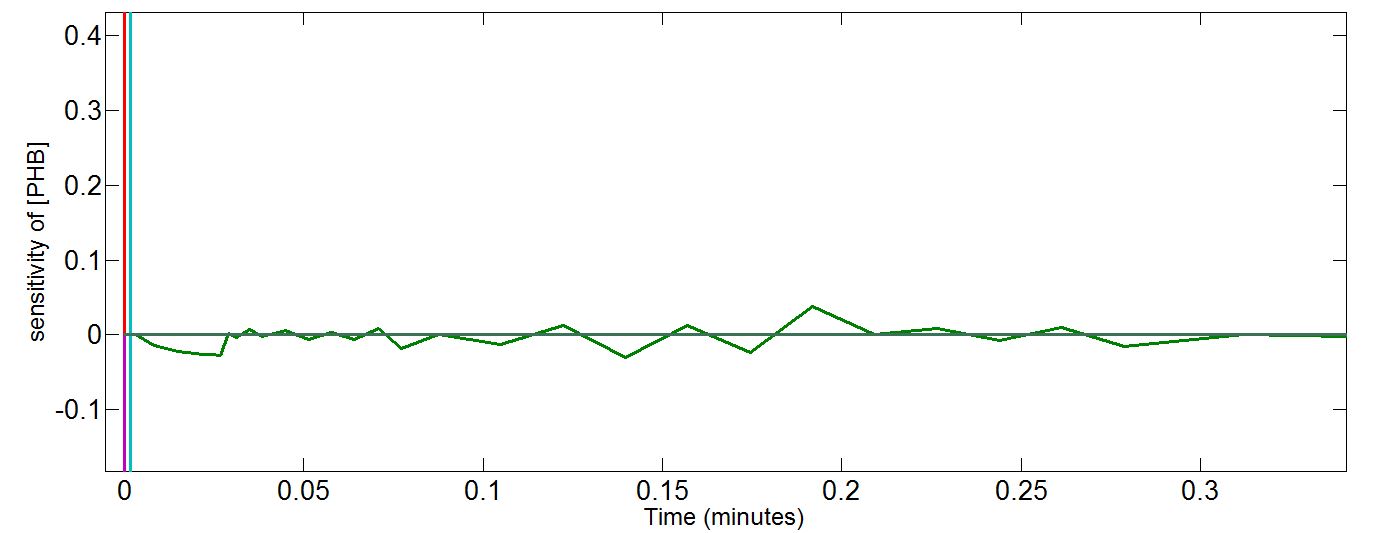


Sensitivity of [PHB]

Time (minutes)


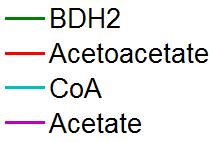


**Others**

According to the simulation result, we found that the PHB production is highly sensitive to the concentration of PhaB and PhaC. In contrast, the system is not sensitive to the concentration of atoAD and atoB. In theory, increase the concentration of any of the enzyme will increase the flux of the system. However, for the strain E.coli K12 MG1655, both atoAD (acetoacetate:acetoacetyl-coA transferase) and atoB （acetoacetyl-coA thiolase) favour the reverse reactions, it becomes the rate limiting step in our pathway. Acetoacetyl-coA intends to be converted to acetoacetate instead of 3HB-coA*^(^*[*^24^*](#_ENREF_24)*^)^*. Therefore, the concentration of PhaB becomes critical for pushing the system to flow in the forward direction.

## Scan with different levels of PhaB

The plot below is to show that increasing PhaB concentration within the engineered organism would have a positive effect on the concentration of the P(3HB) synthesised. As so many parameters within the model were taken from different sources, it would be difficult to give a simulation plot that is accurate quantitatively. It was decided to show the single cell simulation for this because the scans were carried out up to the doubling time of 20mins, hence avoided the issue of scaling-up which would have otherwise subjected the simulation to more errors. Therefore, this plot should be interpreted from a qualitative perspective and the trend should be observed.


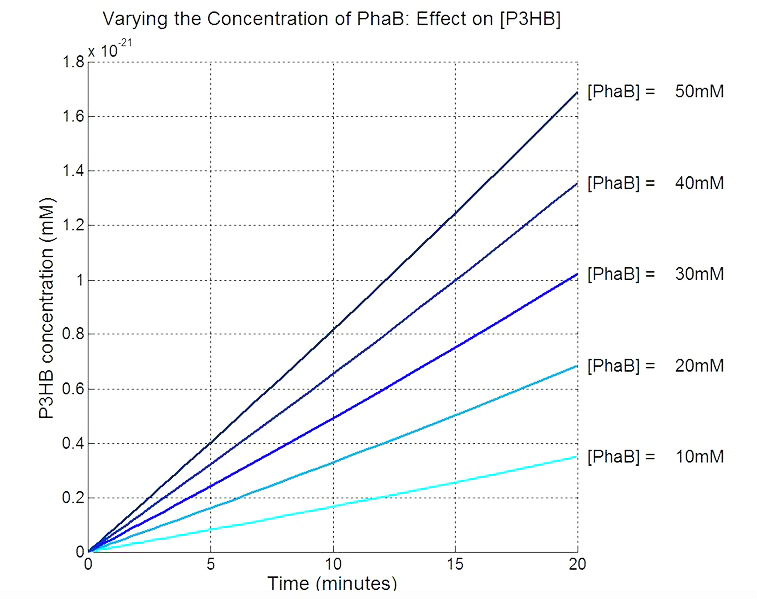


## Difference between promoter expressions after optimisation

In order to choose the strongest promoter that is available to increase the gene expression. We ran a parameter scan of enzyme expression rates of a range of Anderson's constitutive promoters. The simulations shows the PHB production under different Anderson's promoters. The promoters we tested are:

| **promoter** | **biobrick** | **GFP synthesis rate (mM/min)** |
| --- | --- | --- |
| **J23100** | <http://parts.igem.org/Part:BBa_J23100> | 0.41 |
| **J23101** | <http://parts.igem.org/Part:BBa_J23101> | 0.45 |
| **J23104** | <http://parts.igem.org/Part:BBa_J23104> | 0.58 |
| **J23118** | <http://parts.igem.org/Part:BBa_J23118> | 0.31 |

The derivations of the GFP synthesis rate are:

*Relative promoter strengths: J23104 = 1.3RPU, J23101 = 1.0RPU, J23100 = 0.92RPU, J23118 = 0.76RPU *^(^*[*^10^*](#_ENREF_10)*^)^*

- In absolute units: take GFP synthesis rate (molecules per min per cell) and approximate that as a generic protein synthesis rate for the promoter.
  - GFP synthesis rate of 101 = 2232 molecules per min per cell.*^(^*[*^10^*](#_ENREF_10)*^)^*
- Assume 1 molecule in an *E.coli* cell gives a concentration of 1nM.
  - GFP synthesis rate of 101 = 2232 x 1nM = 2.2x10^-6^nM/min per cell
- Plasmid copy number assumed as 200 (as in derivation 1)
  - GFP synthesis rate of 101 in our *E.coli* = 200 x 2.2x10^-6^ = 0.00045nM/min = **0.45mM/min**
  - The GFP synthesis rates of other promoters can be calculated by multiplying the RPU value with the GFP synthesis rate of J23101 promoter.

The simulation result:


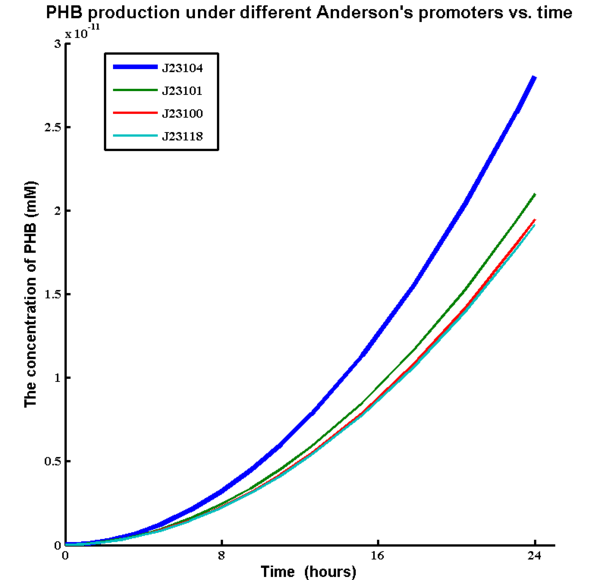


Although there is just a small increase in gene expression rate, J23104 promoter achieved a much higher P(3HB) production rate than any of the other constitutive promoters. Therefore, we decided to use J23104 promoter for our improved biobricks BBa_K1149052 and BBa_K1149051.

# References

1. (2013) MATLAB, Version 8.2.0.701 ed., The MathWorks Inc. <http://www.mathworks.co.uk/>, Natick, MA.

2. Dixon, R. (2011) Designing Predictive Mathematical Models for the Metabolic Pathways Associated with Polyhydroxybutyrate Synthesis in Escherichia coli, Utah State University <http://digitalcommons.usu.edu/cgi/viewcontent.cgi?article=2085&context=etd>.

3. Sezonov, G., Joseleau-Petit, D., and D'Ari, R. (2007) Escherichia coli physiology in Luria-Bertani broth, *J Bacteriol* *189*, 8746-8749.

4. <http://www.geneinfinity.org/sp/sp_dnaprop.html>. (Last accessed October 2013).

5. <http://www.lifetechnologies.com/uk/en/home/references/ambion-tech-support/rna-tools-and-calculators/dna-and-rna-molecular-weights-and-conversions.html>. (Last accessed October 2013).

6. Milo, R., Jorgensen, P., Moran, U., Weber, G., and Springer, M. (2010) BioNumbers--the database of key numbers in molecular and cell biology, *Nucleic Acids Res* *38*, D750-753.

7. <http://www.genscript.com/conversion.html>. (Last accessed October 2013).

8. [http://www.promega.com/~/media/Files/Resources/Technical References/Amino Acid Abbreviations and Molecular Weights.pdf](http://www.promega.com/~/media/Files/Resources/Technical%20References/Amino%20Acid%20Abbreviations%20and%20Molecular%20Weights.pdf). (Last accessed October 2013).

9. <http://www.uniprot.org/uniprot/Q2PEN2&format=html>. (Last accessed October 2013).

10. Hirst, C., Smith, J., Baldwin, G. S., Freemont, P. S., and Kitney, R. I. (2013) AUTOMATED BIOPARTS CHARACTERISATION FOR SYNTHETIC BIOLOGY, *SB6.0 Abstract* <http://sb6.biobricks.org/poster/automated-bioparts-characterisation-for-synthetic-biology/>.

11. Nakashima, K., Ito, K., Nakajima, Y., Yamazawa, R., Miyakawa, S., and Yoshimoto, T. (2009) Closed complex of the D-3-hydroxybutyrate dehydrogenase induced by an enantiomeric competitive inhibitor, *J Biochem* *145*, 467-479.

12. Akil, O., Kebbaj, Z. E., Latruffe, N., and Kebbaj, M. H. S. E. (2009) D-3-hydroxybutyrate oxidation in mitochondria by D-3- Hydroxybutyrate dehydrogenase in Tetrahymena pyriformis, *African Journal of Biochemistry Research*.

13. Sramek, S. J., and Frerman, F. E. (1975) Escherichia coli coenzyme A-transferase: Kinetics, catalytic pathway and structure, *Archives of Biochemistry and Biophysics* *171*, 27-35.

14. <http://www.brenda-enzymes.org/php/result_flat.php4?ecno=2.8.3.8>. (Last accessed October 2013).

15. Hanai, T., Atsumi, S., and Liao, J. C. (2007) Engineered synthetic pathway for isopropanol production in Escherichia coli, *Applied and environmental microbiology* *73*, 7814-7818.

16. Matsumoto, K., Tanaka, Y., Watanabe, T., Motohashi, R., Ikeda, K., Tobitani, K., Yao, M., Tanaka, I., and Taguchi, S. (2013) Directed evolution and structural analysis of NADPH-dependent Acetoacetyl Coenzyme A (Acetoacetyl-CoA) reductase from Ralstonia eutropha reveals two mutations responsible for enhanced kinetics, *Applied and environmental microbiology* *79*, 6134-6139.

17. Ploux, O., Masamune, S., and Walsh, C. T. (1988) The NADPH-linked acetoacetyl-CoA reductase from Zoogloea ramigera. Characterization and mechanistic studies of the cloned enzyme over-produced in Escherichia coli, *Eur J Biochem* *174*, 177-182.

18. Belova, L., Sokolov, A. P., Sidorov, I. A., and Trotsenko, Y. A. (1997) Purification and characterization of NADPH-dependent acetoacetyl-CoA reductase from Methylobacterium extorquens, *FEMS Microbiology Letters* *156*, 275-279.

19. <http://www.brenda-enzymes.org/php/result_flat.php4?ecno=1.1.1.30>. (Last accessed October 2013).

20. Ushimaru, K., Sangiambut, S., Thomson, N., Sivaniah, E., and Tsuge, T. (2013) New insights into activation and substrate recognition of polyhydroxyalkanoate synthase from Ralstonia eutropha, *Applied microbiology and biotechnology* *97*, 1175-1182.

21. Valentin, H. E., and Dennis, D. (1997) Production of poly(3-hydroxybutyrate-co-4-hydroxybutyrate) in recombinant Escherichia coli grown on glucose, *Journal of biotechnology* *58*, 33-38.

22. Hiroe, A., Tsuge, K., Nomura, C. T., Itaya, M., and Tsuge, T. (2012) Rearrangement of gene order in the phaCAB operon leads to effective production of ultrahigh-molecular-weight poly[(R)-3-hydroxybutyrate] in genetically engineered Escherichia coli, *Applied and environmental microbiology* *78*, 3177-3184.

23. <http://www.mathworks.co.uk/help/simbio/ug/calculating-sensitivities.html?nocookie=true%23brbumlrref>. (Last accessed October 2013).

24. Sramek, S. J., and Frerman, F. E. (1977) Steady state kinetic mechanism of the Escherichia coli coenzyme A transferase, *Archives of Biochemistry and Biophysics* *181*, 178-184.
