# Supplementary figures and images for: A Forward-Design Approach to Increase the Production of Poly-3-Hydroxybutyrate in Genetically Engineered Escherichia coli
Source: PLoS One. 2015 Feb 20;10(2):e0117202. doi: 10.1371/journal.pone.0117202 (PMC4336316; doi:10.1371/journal.pone.0117202)

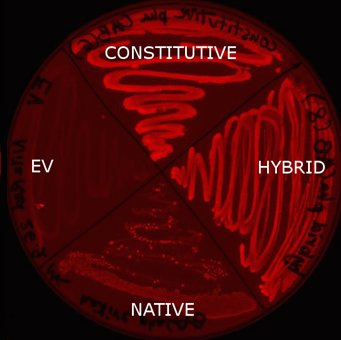

Supplement: S3 Supporting Information — E. coli MG1655 transformed with either empty vector, native, constitutive or hybrid phaCAB constructs were cultured for 24 h at 37°C and 200 rpm shaking (Thermo Scientific MaxQ 6000) in 5 ml LB media, supplemented with 3% glucose (w/v) and 34 μg/mL Chloramphenicol. Liquid cultures were streaked onto LB-agar plates supplemented with 3% glucose (w/v), 34 μg/mL Chloramphenicol and 0.5 μg/ml Nile Red staining (Sigma-Aldrich, MO, USA). Plates were incubated for up to 48 h at 37°C and imaged with a Fuji Film LAS-5000 imager set to 473 nm excitation laser and Cy5 emission filter. (PNG) [file pone.0117202.s003.PNG]

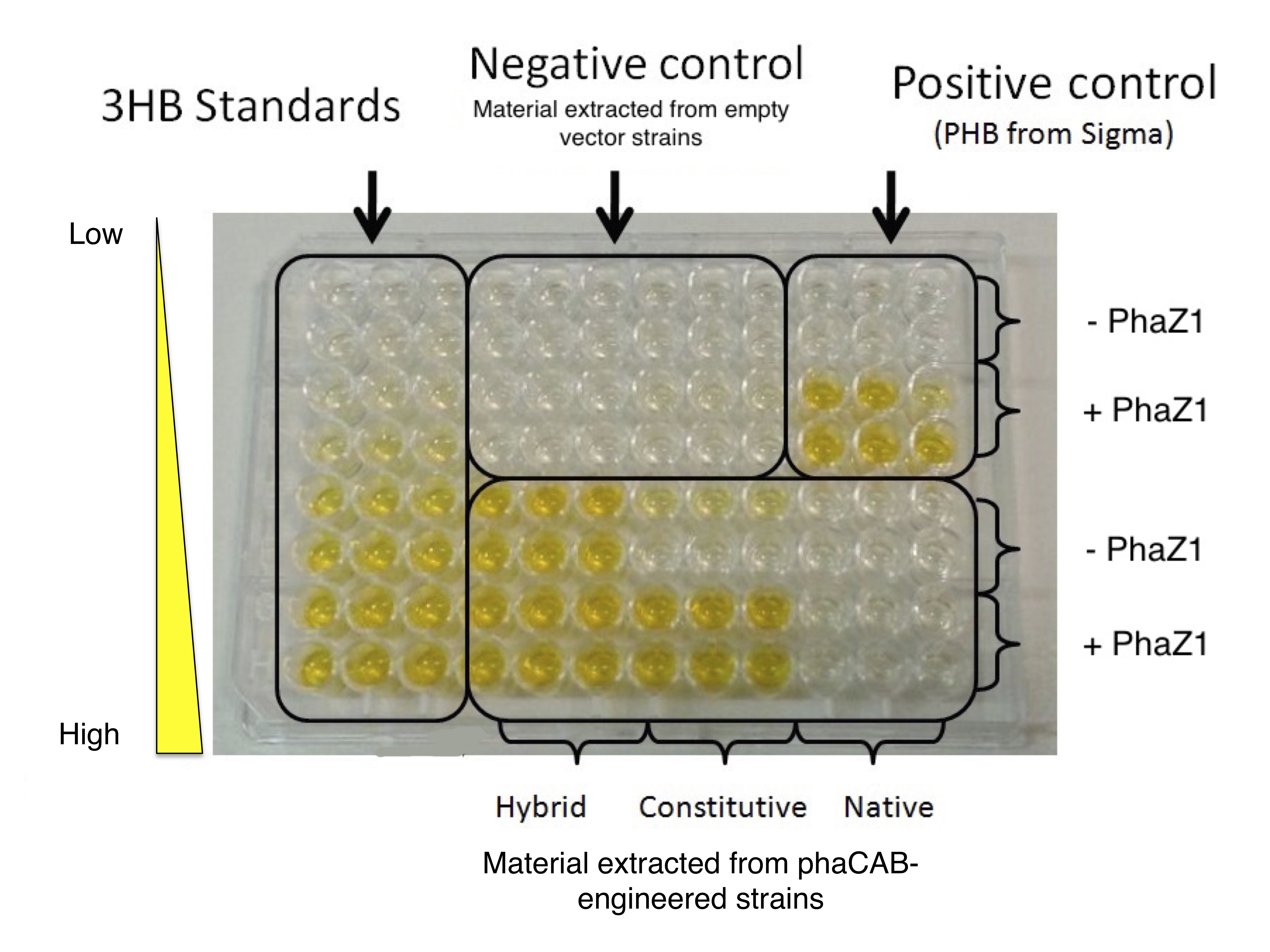

Supplement: S4 Supporting Information — Purified P(3HB) from waste media cultured E. coli MG1655 carrying either empty vector, native phaCAB, constitutive phaCAB or hybrid phaCAB constructs were treated with or without the P(3HB) depolymerase, phaZ1. PhaZ1-treated and untreated samples were analysed with the β-Hydroxybutyrate (Ketone Body) colorimetric assay kit to detect the presence of 3HB, where a yellow colour change indicates the presence of 3HB. (JPG) [file pone.0117202.s004.jpg]

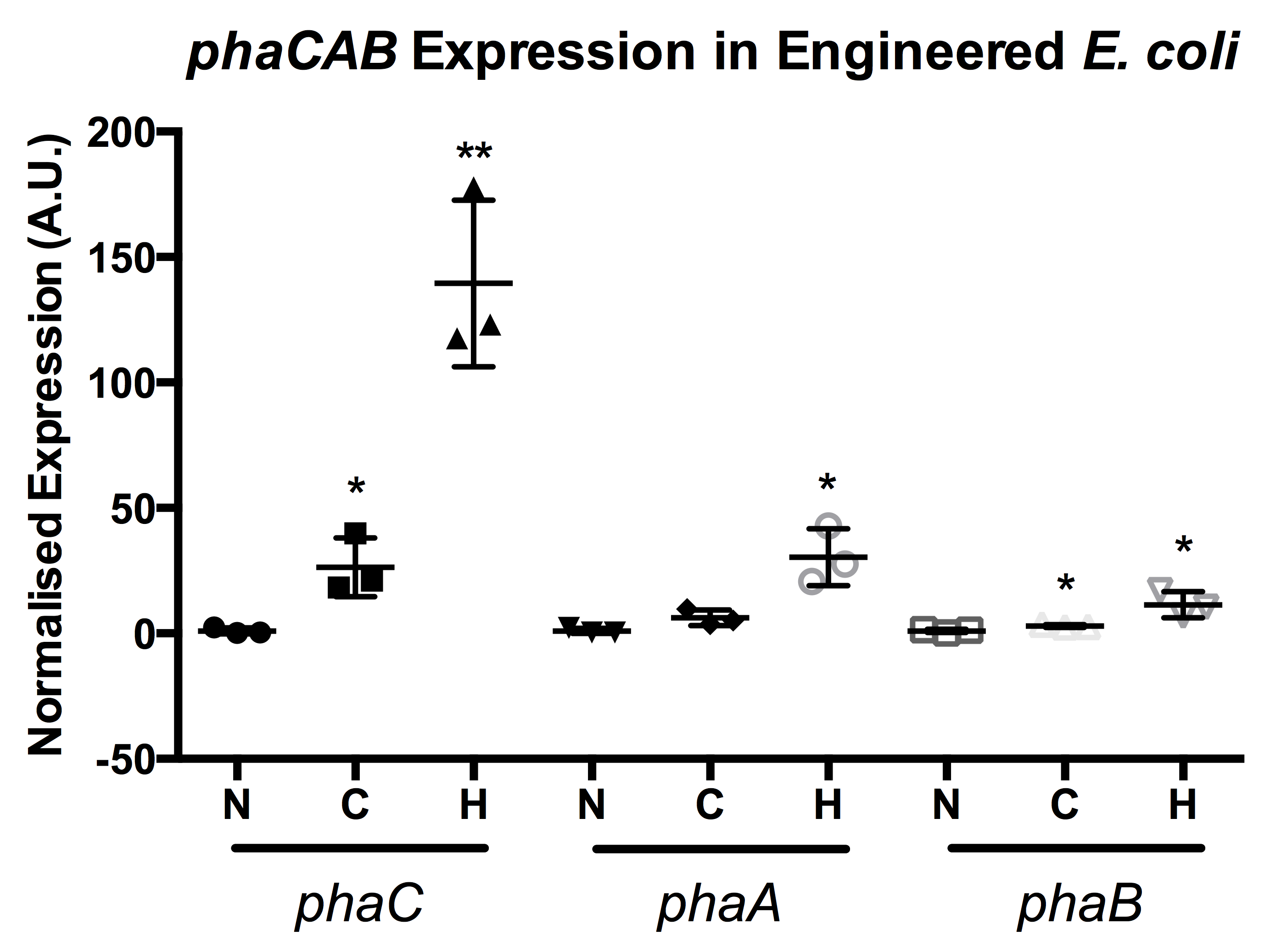

Supplement: S6 Supporting Information — E. coli MG1655 transformed with native [N], constitutive [C] or hybrid [H] phaCAB constructs were cultured in 5 ml LB media, supplemented with 3% glucose (w/v) for 24 h. Analysis of phaCAB gene expression was carried out using qRT-PCR and analyzed via the relative standard curve method. Experiments were carried out in triplicate and were normalized to native phaCAB–engineered E. coli. Error bars, +/- the standard deviation. Student t-test, *P<0.05 and **P <0.01. (TIFF) [file pone.0117202.s006.tiff]
