# Supplementary material for: A Forward-Design Approach to Increase the Production of Poly-3-Hydroxybutyrate in Genetically Engineered Escherichia coli
Source: PLoS One. 2015 Feb 20;10(2):e0117202. doi: 10.1371/journal.pone.0117202 (PMC4336316; doi:10.1371/journal.pone.0117202)
Supplement: S5 Supporting Information — (DOCX) [file pone.0117202.s005.docx]

**A forward-design approach to increase the production of poly-3-hydroxybutyrate in genetically engineered Escherichia coli**

**Supporting Information S5. Engineered phaCAB operon sequences.**

Table of Contents

1. Empty Vector (BBa_K608002) 3

2. Native *phaCAB* operon (BBa_K934001) 4

3. Constitutive *phaCAB* operon (BBa_K1149052) 6

4. Hybrid *phaCAB* operon (BBa_K1149051) 8

# 1. Empty Vector (BBa_K608002)

**Description:** Control construct consisting of J23104 and RBS B0034 but no insert (i.e. does not contain a *phaCAB* operon).

**Plasmid backbone:** pSB1C3


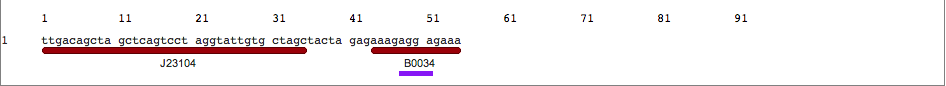


>BBa_K608002 Part-only sequence (55 bp) ttgacagctagctcagtcctaggtattgtgctagctactagagaaagaggagaaa

# 2. Native *phaCAB* operon (BBa_K934001)

**Description:** Native *phaCAB* operon from *Ralstonia eutropha* H16.

**Plasmid backbone:** pSB1C3


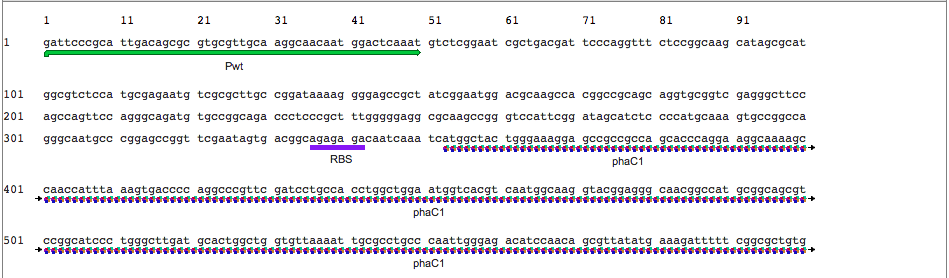
Screenshot displays first 600 bases.

>BBa_K934001 Part-only sequence (4208 bp) gattcccgcattgacagcgcgtgcgttgcaaggcaacaatggactcaaatgtctcggaatcgctgacgattcccaggtttctccggcaagcatagcgcatggcgtctccatgcgagaatgtcgcgcttgccggataaaaggggagccgctatcggaatggacgcaagccacggccgcagcaggtgcggtcgagggcttccagccagttccagggcagatgtgccggcagaccctcccgctttgggggaggcgcaagccgggtccattcggatagcatctccccatgcaaagtgccggcca gggcaatgcccggagccggttcgaatagtgacggcagagagacaatcaaatcatggctactgggaaaggagccgccgccagcacccaggaaggcaaaagccaaccatttaaagtgaccccaggcccgttcgatcctgccacctggctggaatggtcacgtcaatggcaaggtacggagggcaacggccatgcggcagcgtccggcatccctgggcttgatgcactggctggtgttaaaattgcgcctgcccaattgggagacatccaacagcgttatatgaaagatttttcggcgctgtg gcaggcgatggcggaaggtaaagcagaggcgacgggaccgctgcatgaccgtcgctttgctggcgacgcctggcgtaccaatttgccctatcgttttgcggcagccttctacctgctgaacgctcgtgccctgactgagctggcagatgctgtcgaagcggatgccaaaacccgccaacgcatccgttttgccatctcacagtgggtcgacgcaatgtcaccggccaacttcctggccaccaatccggaagcacagcgcctcctgattgaaagcggcggggaatcgctgcgcgccggcgt tcgtaacatgatggaagatctgacccgtggtaagatttcgcagacggatgagtcggccttcgaggtgggtcgcaatgttgcagtgacagaaggcgcggtggtgtttgagaacgaatactttcaacttctccagtataaaccactgacggataaagtgcatgcgcgcccattgttaatggttcccccgtgcattaataaatactatatcctggatcttcagccggaaagctccttagtgcgccacgtggtggaacaggggcatactgtgtttttagttagctggcgcaatccagatgcctc aatggccggcagtacctgggatgactacattgaacacgcggctattcgcgcgatcgaagtggcacgcgacatctcaggtcaggacaagattaacgtactcggcttttgcgtcggtggcactatcgtgagcaccgctctggcggtactggctgcgcgtggtgaacaccccgcagcgtcggttacgcttctgaccacactgttggatttcgccgacactggcatcttggatgtatttgtcgatgagggccacgttcagctgcgcgaggccactctgggtggcggcgccggcgcaccgtgcgc tctgctgcgcggtctggagctcgcgaatactttttctttcttacgcccgaacgatcttgtctggaattacgtagttgacaactatctcaaaggtaacacgcccgtgccgtttgatttattattctggaatggggatgcgaccaacctgccgggtccttggtattgttggtatctccgtcatacatacttacagaatgaattaaaggtcccaggtaaacttactgtgtgtggtgtcccggtagatctggcgagtatcgacgtgccgacctacatttatgggagtcgtgaagatcatattgt gccgtggaccgccgcgtacgcttctaccgcgcttctggcaaacaaattacgtttcgttctgggtgccagcggccacattgcgggtgttattaatcctccggccaaaaataaacgctcgcattggacaaacgacgccttgccggagagccctcagcagtggttggcgggtgccattgagcaccatggaagttggtggccggactggacagcctggctggcgggccaagcaggcgcaaaacgcgctgcgccagccaactatggcaacgcgcgctaccgcgcgattgaacccgcgcctggccg ctatgtaaaagcaaaagcgtgacgcttgcatgagtgccggcgtgcgtcatgcacggcgccggcaggcctgcacgttccctcccgtttccattgaaaggactacacaatgaccgatgttgttatcgtcagtgccgcgcgcaccgccgtgggtaaatttggcggcagtctggcgaaaatcccggcgccagaattgggcgcggtggtaatcaaagcggccctggaacgcgccggtgtgaaacctgagcaggtgagcgaagtaattatgggtcaggttctgaccgcgggcagcgggcaaaatcc ggcgcgtcaggctgcgattaaggcaggccttccggcgatggtgcccgcgatgaccattaacaaagtatgcggatcgggactgaaagcagtgatgctggccgctaacgccattatggcaggcgacgcggaaattgtggtcgctggcggacaggagaatatgtcagccgcgcctcatgtgctgccgggtagccgcgacggctttcgcatgggtgatgcaaaactggtcgatactatgattgttgacggcttatgggatgtgtacaaccagtaccacatgggcatcaccgccgagaacgtggc caaggaatacggcatcacacgcgaggcgcaggatgagttcgccgtcggctcgcagaacaaggccgaagccgcgcagaaggccggcatgtttgacgaagagatcgtcccggtgctgatcccgcagcgcaagggcgacccggtggccttcaagaccgacgagttcgtgcgccagggcgccacgctggacagcatgtccggcctcaagcccgccttcgacaaggccggcacggtgaccgcggccaacgcctcgggcctgaacgacggcgccgccgcggtggtggtgatgtcggcggccaaggc caaggaactgggcctgaccccgctggccacgatcaagagctatgccaacgccggtgtcgatcccaaggtgatgggcatgggcccggtgccggcctccaagcgcgccctgtcgcgcgccgagtggaccccgcaagacctggacctgatggagatcaacgaggcctttgccgcgcaggcgctggcggtgcaccagcagatgggctgggacacctccaaggtcaatgtgaacggcggcgccatcgccatcggccacccgatcggcgcgtcgggctgccgtatcctggtgacgctgctgcacga gatgaagcgccgtgacgcgaagaagggcctggcctcgctgtgcatcggcggcggcatgggcgtggcgctggcagtcgagcgcaaataaggaaggggttttccggggccgcgcgcggttggcgcggacccggcgacgataacgaagccaatcaaggagtggacatgactcagcgcattgcgtatgtgaccggcggcatgggtggtatcggaaccgccatttgccagcggctggccaaggatggctttcgtgtggtggccggttgcggccccaactcgccgcgccgcgaaaagtggctggag cagcagaaggccctgggcttcgatttcattgcctcggaaggcaatgtggctgactgggactcgaccaagaccgcattcgacaaggtcaagtccgaggtcggcgaggttgatgtgctgatcaacaacgccggtatcacccgcgacgtggtgttccgcaagatgacccgcgccgactgggatgcggtgatcgacaccaacctgacctcgctgttcaacgtcaccaagcaggtgatcgacggcatggccgaccgtggctggggccgcatcgtcaacatctcgtcggtgaacgggcagaagggc cagttcggccagaccaactactccaccgccaaggccggcctgcatggcttcaccatggcactggcgcaggaagtggcgaccaagggcgtgaccgtcaacacggtctctccgggctatatcgccaccgacatggtcaaggcgatccgccaggacgtgctcgacaagatcgtcgcgacgatcccggtcaagcgcctgggcctgccggaagagatcgcctcgatctgcgcctggttgtcgtcggaggagtccggtttctcgaccggcgccgacttctcgctcaacggcggcctgcatatgggc tgacctgc

# 3. Constitutive *phaCAB* operon (BBa_K1149052)

**Description:** Constitutive *phaCAB* operon, where the native promoter and RBS have been replaced with J23104 and RBS B0034.

**Plasmid backbone:** pSB1C3.


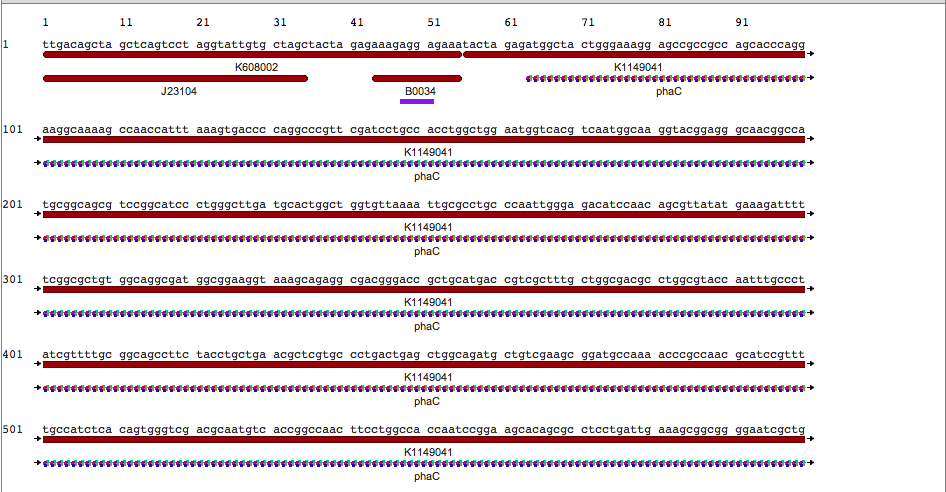
Screenshot displays first 600 bases.

>BBa_K1149052 Part-only sequence (3911 bp) ttgacagctagctcagtcctaggtattgtgctagctactagagaaagaggagaaatactagagatggctactgggaaaggagccgccgccagcacccaggaaggcaaaagccaaccatttaaagtgaccccaggcccgttcgatcctgccacctggctggaatggtcacgtcaatggcaaggtacggagggcaacggccatgcggcagcgtccggcatccctgggcttgatgcactggctggtgttaaaattgcgcctgcccaattgggagacatccaacagcgttatatgaaagatttt tcggcgctgtggcaggcgatggcggaaggtaaagcagaggcgacgggaccgctgcatgaccgtcgctttgctggcgacgcctggcgtaccaatttgccctatcgttttgcggcagccttctacctgctgaacgctcgtgccctgactgagctggcagatgctgtcgaagcggatgccaaaacccgccaacgcatccgttttgccatctcacagtgggtcgacgcaatgtcaccggccaacttcctggccaccaatccggaagcacagcgcctcctgattgaaagcggcggggaatcgctg cgcgccggcgttcgtaacatgatggaagatctgacccgtggtaagatttcgcagacggatgagtcggccttcgaggtgggtcgcaatgttgcagtgacagaaggcgcggtggtgtttgagaacgaatactttcaacttctccagtataaaccactgacggataaagtgcatgcgcgcccattgttaatggttcccccgtgcattaataaatactatatcctggatcttcagccggaaagctccttagtgcgccacgtggtggaacaggggcatactgtgtttttagttagctggcgcaat ccagatgcctcaatggccggcagtacctgggatgactacattgaacacgcggctattcgcgcgatcgaagtggcacgcgacatctcaggtcaggacaagattaacgtactcggcttttgcgtcggtggcactatcgtgagcaccgctctggcggtactggctgcgcgtggtgaacaccccgcagcgtcggttacgcttctgaccacactgttggatttcgccgacactggcatcttggatgtatttgtcgatgagggccacgttcagctgcgcgaggccactctgggtggcggcgccggc gcaccgtgcgctctgctgcgcggtctggagctcgcgaatactttttctttcttacgcccgaacgatcttgtctggaattacgtagttgacaactatctcaaaggtaacacgcccgtgccgtttgatttattattctggaatggggatgcgaccaacctgccgggtccttggtattgttggtatctccgtcatacatacttacagaatgaattaaaggtcccaggtaaacttactgtgtgtggtgtcccggtagatctggcgagtatcgacgtgccgacctacatttatgggagtcgtgaa gatcatattgtgccgtggaccgccgcgtacgcttctaccgcgcttctggcaaacaaattacgtttcgttctgggtgccagcggccacattgcgggtgttattaatcctccggccaaaaataaacgctcgcattggacaaacgacgccttgccggagagccctcagcagtggttggcgggtgccattgagcaccatggaagttggtggccggactggacagcctggctggcgggccaagcaggcgcaaaacgcgctgcgccagccaactatggcaacgcgcgctaccgcgcgattgaaccc gcgcctggccgctatgtaaaagcaaaagcgtgacgcttgcatgagtgccggcgtgcgtcatgcacggcgccggcaggcctgcacgttccctcccgtttccattgaaaggactacacaatgaccgatgttgttatcgtcagtgccgcgcgcaccgccgtgggtaaatttggcggcagtctggcgaaaatcccggcgccagaattgggcgcggtggtaatcaaagcggccctggaacgcgccggtgtgaaacctgagcaggtgagcgaagtaattatgggtcaggttctgaccgcgggcagc gggcaaaatccggcgcgtcaggctgcgattaaggcaggccttccggcgatggtgcccgcgatgaccattaacaaagtatgcggatcgggactgaaagcagtgatgctggccgctaacgccattatggcaggcgacgcggaaattgtggtcgctggcggacaggagaatatgtcagccgcgcctcatgtgctgccgggtagccgcgacggctttcgcatgggtgatgcaaaactggtcgatactatgattgttgacggcttatgggatgtgtacaaccagtaccacatgggcatcaccgcc gagaacgtggccaaggaatacggcatcacacgcgaggcgcaggatgagttcgccgtcggctcgcagaacaaggccgaagccgcgcagaaggccggcatgtttgacgaagagatcgtcccggtgctgatcccgcagcgcaagggcgacccggtggccttcaagaccgacgagttcgtgcgccagggcgccacgctggacagcatgtccggcctcaagcccgccttcgacaaggccggcacggtgaccgcggccaacgcctcgggcctgaacgacggcgccgccgcggtggtggtgatgtcg gcggccaaggccaaggaactgggcctgaccccgctggccacgatcaagagctatgccaacgccggtgtcgatcccaaggtgatgggcatgggcccggtgccggcctccaagcgcgccctgtcgcgcgccgagtggaccccgcaagacctggacctgatggagatcaacgaggcctttgccgcgcaggcgctggcggtgcaccagcagatgggctgggacacctccaaggtcaatgtgaacggcggcgccatcgccatcggccacccgatcggcgcgtcgggctgccgtatcctggtgacg ctgctgcacgagatgaagcgccgtgacgcgaagaagggcctggcctcgctgtgcatcggcggcggcatgggcgtggcgctggcagtcgagcgcaaataaggaaggggttttccggggccgcgcgcggttggcgcggacccggcgacgataacgaagccaatcaaggagtggacatgactcagcgcattgcgtatgtgaccggcggcatgggtggtatcggaaccgccatttgccagcggctggccaaggatggctttcgtgtggtggccggttgcggccccaactcgccgcgccgcgaaa agtggctggagcagcagaaggccctgggcttcgatttcattgcctcggaaggcaatgtggctgactgggactcgaccaagaccgcattcgacaaggtcaagtccgaggtcggcgaggttgatgtgctgatcaacaacgccggtatcacccgcgacgtggtgttccgcaagatgacccgcgccgactgggatgcggtgatcgacaccaacctgacctcgctgttcaacgtcaccaagcaggtgatcgacggcatggccgaccgtggctggggccgcatcgtcaacatctcgtcggtgaacg ggcagaagggccagttcggccagaccaactactccaccgccaaggccggcctgcatggcttcaccatggcactggcgcaggaagtggcgaccaagggcgtgaccgtcaacacggtctctccgggctatatcgccaccgacatggtcaaggcgatccgccaggacgtgctcgacaagatcgtcgcgacgatcccggtcaagcgcctgggcctgccggaagagatcgcctcgatctgcgcctggttgtcgtcggaggagtccggtttctcgaccggcgccgacttctcgctcaacggcggcc tgcatatgggc

# 4. Hybrid *phaCAB* operon (BBa_K1149051)

**Description:** Hybrid *phaCAB* operon; the hybrid operon design was constructed in parallel to the constitutive operon and is noted for its dual promoter and RBS combinations; J23104 RBS B0034, native promoter and native RBS.

**Plasmid backbone:** pSB1C3.


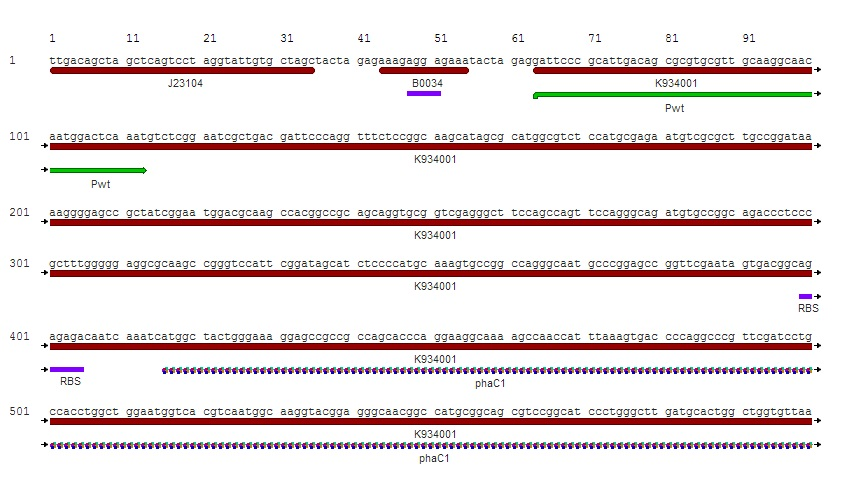
Screenshot displays first 600 bases.

>BBa_K1149051 Part-only sequence (4271 bp) ttgacagctagctcagtcctaggtattgtgctagctactagagaaagaggagaaatactagaggattcccgcattgacagcgcgtgcgttgcaaggcaacaatggactcaaatgtctcggaatcgctgacgattcccaggtttctccggcaagcatagcgcatggcgtctccatgcgagaatgtcgcgcttgccggataaaaggggagccgctatcggaatggacgcaagccacggccgcagcaggtgcggtcgagggcttccagccagttccagggcagatgtgccggcagaccctccc gctttgggggaggcgcaagccgggtccattcggatagcatctccccatgcaaagtgccggccagggcaatgcccggagccggttcgaatagtgacggcagagagacaatcaaatcatggctactgggaaaggagccgccgccagcacccaggaaggcaaaagccaaccatttaaagtgaccccaggcccgttcgatcctgccacctggctggaatggtcacgtcaatggcaaggtacggagggcaacggccatgcggcagcgtccggcatccctgggcttgatgcactggctggtgttaa aattgcgcctgcccaattgggagacatccaacagcgttatatgaaagatttttcggcgctgtggcaggcgatggcggaaggtaaagcagaggcgacgggaccgctgcatgaccgtcgctttgctggcgacgcctggcgtaccaatttgccctatcgttttgcggcagccttctacctgctgaacgctcgtgccctgactgagctggcagatgctgtcgaagcggatgccaaaacccgccaacgcatccgttttgccatctcacagtgggtcgacgcaatgtcaccggccaacttcctggc caccaatccggaagcacagcgcctcctgattgaaagcggcggggaatcgctgcgcgccggcgttcgtaacatgatggaagatctgacccgtggtaagatttcgcagacggatgagtcggccttcgaggtgggtcgcaatgttgcagtgacagaaggcgcggtggtgtttgagaacgaatactttcaacttctccagtataaaccactgacggataaagtgcatgcgcgcccattgttaatggttcccccgtgcattaataaatactatatcctggatcttcagccggaaagctccttagt gcgccacgtggtggaacaggggcatactgtgtttttagttagctggcgcaatccagatgcctcaatggccggcagtacctgggatgactacattgaacacgcggctattcgcgcgatcgaagtggcacgcgacatctcaggtcaggacaagattaacgtactcggcttttgcgtcggtggcactatcgtgagcaccgctctggcggtactggctgcgcgtggtgaacaccccgcagcgtcggttacgcttctgaccacactgttggatttcgccgacactggcatcttggatgtatttgt cgatgagggccacgttcagctgcgcgaggccactctgggtggcggcgccggcgcaccgtgcgctctgctgcgcggtctggagctcgcgaatactttttctttcttacgcccgaacgatcttgtctggaattacgtagttgacaactatctcaaaggtaacacgcccgtgccgtttgatttattattctggaatggggatgcgaccaacctgccgggtccttggtattgttggtatctccgtcatacatacttacagaatgaattaaaggtcccaggtaaacttactgtgtgtggtgtccc ggtagatctggcgagtatcgacgtgccgacctacatttatgggagtcgtgaagatcatattgtgccgtggaccgccgcgtacgcttctaccgcgcttctggcaaacaaattacgtttcgttctgggtgccagcggccacattgcgggtgttattaatcctccggccaaaaataaacgctcgcattggacaaacgacgccttgccggagagccctcagcagtggttggcgggtgccattgagcaccatggaagttggtggccggactggacagcctggctggcgggccaagcaggcgcaaa acgcgctgcgccagccaactatggcaacgcgcgctaccgcgcgattgaacccgcgcctggccgctatgtaaaagcaaaagcgtgacgcttgcatgagtgccggcgtgcgtcatgcacggcgccggcaggcctgcacgttccctcccgtttccattgaaaggactacacaatgaccgatgttgttatcgtcagtgccgcgcgcaccgccgtgggtaaatttggcggcagtctggcgaaaatcccggcgccagaattgggcgcggtggtaatcaaagcggccctggaacgcgccggtgtgaa acctgagcaggtgagcgaagtaattatgggtcaggttctgaccgcgggcagcgggcaaaatccggcgcgtcaggctgcgattaaggcaggccttccggcgatggtgcccgcgatgaccattaacaaagtatgcggatcgggactgaaagcagtgatgctggccgctaacgccattatggcaggcgacgcggaaattgtggtcgctggcggacaggagaatatgtcagccgcgcctcatgtgctgccgggtagccgcgacggctttcgcatgggtgatgcaaaactggtcgatactatgat tgttgacggcttatgggatgtgtacaaccagtaccacatgggcatcaccgccgagaacgtggccaaggaatacggcatcacacgcgaggcgcaggatgagttcgccgtcggctcgcagaacaaggccgaagccgcgcagaaggccggcatgtttgacgaagagatcgtcccggtgctgatcccgcagcgcaagggcgacccggtggccttcaagaccgacgagttcgtgcgccagggcgccacgctggacagcatgtccggcctcaagcccgccttcgacaaggccggcacggtgaccgc ggccaacgcctcgggcctgaacgacggcgccgccgcggtggtggtgatgtcggcggccaaggccaaggaactgggcctgaccccgctggccacgatcaagagctatgccaacgccggtgtcgatcccaaggtgatgggcatgggcccggtgccggcctccaagcgcgccctgtcgcgcgccgagtggaccccgcaagacctggacctgatggagatcaacgaggcctttgccgcgcaggcgctggcggtgcaccagcagatgggctgggacacctccaaggtcaatgtgaacggcggcgc catcgccatcggccacccgatcggcgcgtcgggctgccgtatcctggtgacgctgctgcacgagatgaagcgccgtgacgcgaagaagggcctggcctcgctgtgcatcggcggcggcatgggcgtggcgctggcagtcgagcgcaaataaggaaggggttttccggggccgcgcgcggttggcgcggacccggcgacgataacgaagccaatcaaggagtggacatgactcagcgcattgcgtatgtgaccggcggcatgggtggtatcggaaccgccatttgccagcggctggccaag gatggctttcgtgtggtggccggttgcggccccaactcgccgcgccgcgaaaagtggctggagcagcagaaggccctgggcttcgatttcattgcctcggaaggcaatgtggctgactgggactcgaccaagaccgcattcgacaaggtcaagtccgaggtcggcgaggttgatgtgctgatcaacaacgccggtatcacccgcgacgtggtgttccgcaagatgacccgcgccgactgggatgcggtgatcgacaccaacctgacctcgctgttcaacgtcaccaagcaggtgatcgac ggcatggccgaccgtggctggggccgcatcgtcaacatctcgtcggtgaacgggcagaagggccagttcggccagaccaactactccaccgccaaggccggcctgcatggcttcaccatggcactggcgcaggaagtggcgaccaagggcgtgaccgtcaacacggtctctccgggctatatcgccaccgacatggtcaaggcgatccgccaggacgtgctcgacaagatcgtcgcgacgatcccggtcaagcgcctgggcctgccggaagagatcgcctcgatctgcgcctggttgtcg tcggaggagtccggtttctcgaccggcgccgacttctcgctcaacggcggcctgcatatgggctgacctgc
